# Vitamin D_3_ supplementation during pregnancy and lactation for women living with HIV in Tanzania: A randomized controlled trial

**DOI:** 10.1371/journal.pmed.1003973

**Published:** 2022-04-15

**Authors:** Christopher R. Sudfeld, Karim P. Manji, Alfa Muhihi, Christopher P. Duggan, Said Aboud, Fadhlun M. Alwy Al-Beity, Molin Wang, Ning Zhang, Nzovu Ulenga, Wafaie W. Fawzi

**Affiliations:** 1 Department of Global Health and Population, Harvard T.H. Chan School of Public Health, Boston, Massachusetts, United States of America; 2 Department of Nutrition, Harvard T.H. Chan School of Public Health, Boston, Massachusetts, United States of America; 3 Department of Pediatrics and Child Health, Muhimbili University of Health and Allied Sciences, Dar es Salaam, Tanzania; 4 Management and Development for Health, Dar es Salaam, Tanzania; 5 Division of Gastroenterology, Hepatology, and Nutrition, Boston Children’s Hospital and Harvard Medical School, Boston, Massachusetts, United States of America; 6 Department of Microbiology and Immunology, Muhimbili University of Health and Allied Sciences, Dar es Salaam, Tanzania; 7 Department of Obstetrics and Gynecology, Muhimbili University of Health and Allied Sciences, Dar es Salaam, Tanzania; 8 Department of Biostatistics, Harvard T.H. Chan School of Public Health, Boston, Massachusetts, United States of America; 9 Department of Epidemiology, Harvard T.H. Chan School of Public Health, Boston, Massachusetts, United States of America; 10 Channing Division of Network Medicine, Brigham and Women’s Hospital and Harvard Medical School, Boston, Massachusetts, United States of America; The University of Edinburgh Usher Institute of Population Health Sciences and Informatics, UNITED KINGDOM

## Abstract

**Background:**

Observational studies suggest that vitamin D deficiency among people living with HIV is associated with a greater risk of disease progression and death. Low levels of vitamin D in pregnancy are also associated with poor fetal and infant growth. Therefore, vitamin D supplementation may improve clinical outcomes for pregnant women living with HIV and improve fetal and postnatal growth for their infants.

**Methods and findings:**

We conducted a randomized, triple-blind, placebo-controlled trial of vitamin D_3_ supplementation among pregnant and lactating women living with HIV in Dar es Salaam, Tanzania (ClinicalTrials.gov NCT02305927). Participants were randomized with 1:1 allocation stratified by study clinic to receive either daily 3,000 IU vitamin D_3_ supplements or matching placebo supplements from the second trimester of pregnancy (12–27 weeks) until 1 year postpartum. The primary outcomes were (i) maternal HIV progression or death, (ii) small-for-gestational-age (SGA) live births (<10th percentile), and (iii) infant stunting at 1 year of age (length-for-age *z-*score < −2). We also examined the effect of vitamin D_3_ supplementation on secondary maternal and infant health outcomes, maternal and infant serum 25-hydroxyvitamin D (25[OH]D) concentrations, and maternal hypercalcemia. An intent-to-treat analysis was used as the primary analytic approach. We enrolled 2,300 pregnant women between June 15, 2015, and April 17, 2018, and follow-up of mothers and infants was completed on October 20, 2019. There were 1,148 pregnant women randomly assigned to the vitamin D_3_ group, and 1,152 to the placebo group. The proportion of mothers lost to follow-up at 1 year postpartum was 6.6% in the vitamin D_3_ group (83 of 1,148) and 6.6% in the placebo group (76 of 1,152). The proportion of children lost to follow-up at 1 year of age was 5.5% in the vitamin D_3_ group (59 of 1,074 live births) and 5.2% in the placebo group (57 of 1,093 live births). There was no difference in the risk of maternal HIV progression or death, with 166 events during 1,461 person-years of follow-up in the vitamin D_3_ group and 141 events during 1,469 person-years of follow-up in the placebo group (hazard ratio 1.21, 95% CI 0.97 to 1.52, *p =* 0.09). There was no difference in the risk of SGA birth between the vitamin D_3_ (229 SGA births among 1,070 live births) and placebo groups (236 SGA births among 1,091 live births) (relative risk 1.03, 95% CI 0.87 to 1.22, *p =* 0.70). There was also no difference in the risk of infant stunting at 1 year of age between the vitamin D_3_ (407 events among 867 infants) and placebo groups (413 events among 873 infants) (relative risk 1.00, 95% CI 0.92 to 1.10, *p =* 0.95). In terms of adverse events, no cases of maternal hypercalcemia were identified. One hypersensitivity reaction to the trial supplements occurred for a pregnant woman in the placebo group. A limitation of our study is that our findings may not be generalizable to HIV-negative pregnant women or contexts where severe vitamin D deficiency is prevalent.

**Conclusions:**

The trial findings do not support routine vitamin D supplementation for pregnant and lactating women living with HIV in Tanzania.

**Trial registration:**

ClinicalTrials.gov Identifier: NCT02305927.

## Abstract 2

Christopher R. Sudfeld and colleagues, investigate the impact of vitamin D supplementation on clinical outcomes for pregnant women living with HIV, and growth outcomes for their infants.

## Author summary

**Why was this study done?:**

- Observational studies have found that vitamin D deficiency among people living with HIV is associated with an increased risk of HIV disease progression and death.
- Low levels of vitamin D in pregnancy are also associated with adverse birth outcomes and poor infant growth.

**What did the researchers do and find?:**

- We conducted a randomized controlled trial of maternal vitamin D_3_ supplementation during pregnancy and lactation and found no effect on the primary trial outcomes of maternal HIV progression or death, small-for-gestational-age (SGA) live births, and infant stunting (length-for-age *z-*score < −2) at 1 year of age.
- There was no effect on most secondary outcomes; however, we found that vitamin D supplementation decreased the risk of death for mothers and increased the risk of preterm birth.
- Maternal vitamin D supplementation increased maternal serum 25-hydroxyvitamin D (25[OH]D) concentrations throughout follow-up and increased infant 25(OH)D at 6 weeks and 6 months of age, but not at 12 months of age.

**What do these findings mean?:**

- The trial findings are not in support of routine vitamin D supplementation for pregnant and lactating women living with HIV.
- The mixed findings on the secondary outcomes of maternal death and preterm birth should be interpreted with caution and require replication in other randomized trials.

## Introduction

Implementation of effective perinatal and postnatal interventions for the prevention of mother-to-child transmission of HIV (PMTCT), including triple-drug antiretroviral therapy (ART) during pregnancy and lactation, has substantially reduced the incidence of pediatric HIV infections globally [1,2]. However, this success has also led to an increasing number of HIV-exposed uninfected (HEU) children, who have a mother living with HIV but are not infected themselves [1]. It is estimated that in 2018 there were 14.8 million HEU children globally, of whom 13.2 million (approximately 90%) resided in sub-Saharan Africa [3]. While ART substantially reduces the risk of mother-to-child transmission of HIV, studies have indicated that pregnant women on ART remain at increased risk for adverse birth outcomes—including low birthweight (LBW), preterm birth, and small-for-gestational-age (SGA) birth—compared to HIV-uninfected pregnant women [4,5]. There is also observational evidence that HEU children are at increased risk of morbidity, growth faltering, and mortality compared to children born to HIV-uninfected mothers [6–8]. Consequently, interventions to improve birth outcomes and support the broader health, growth, and development of children born to women living with HIV are needed, particularly in sub-Saharan Africa.

Vitamin D is known to be essential for calcium homeostasis and bone health, but there is growing evidence of immunomodulatory and other extraskeletal effects [9–11]. Observational evidence indicates that vitamin D deficiency, as assessed by serum or plasma concentration of 25-hydroxyvitamin D (25[OH]D), is common among adults living with HIV and is associated with increased risk of HIV disease progression and mortality [12–14]. Vitamin D deficiency in pregnancy, regardless of maternal HIV status, has also been shown to be associated with increased risk of prematurity, LBW, SGA birth, and child linear growth faltering [15–17]. Cohort studies of Tanzanian pregnant women living with HIV found that low 25(OH)D concentrations were associated with increased risk of maternal HIV disease progression, anemia, wasting, and child respiratory infections [14,18,19]. Nevertheless, the efficacy of vitamin D supplementation during pregnancy and lactation on birth outcomes and infant growth in randomized controlled trials remains inconclusive [20,21]. Further, to our knowledge no vitamin D supplementation trials have been conducted among pregnant women living with HIV.

We conducted a randomized, triple-blind, placebo-controlled trial of vitamin D_3_ supplementation during pregnancy and lactation for women living with HIV in Tanzania. The primary trial outcomes were maternal HIV progression or death, SGA live births, and infant stunting at 12 months of age. We also examined the effect of the vitamin D_3_ supplementation on several secondary maternal and infant health outcomes, as well as maternal and infant serum 25(OH)D concentrations and maternal hypercalcemia as a targeted safety outcome.

## Methods

### Study design

We conducted a randomized, parallel-group, triple-blind, placebo-controlled trial of vitamin D_3_ supplementation for pregnant women living with HIV in urban Dar es Salaam, Tanzania (S1 Protocol). The trial protocol was published previously [22]. We enrolled participants at 5 public antenatal care clinics that provided care for pregnant women living with HIV with the Option B+ approach, where all pregnant women living with HIV initiated lifelong triple-drug ART, irrespective of clinical or immunologic status. Pregnant women living with HIV were enrolled in the trial between June 15, 2015, and April 17, 2018; follow-up of all mothers and infants was completed on October 20, 2019. Biomarker analyses were delayed by the COVID-19 pandemic but were completed in Boston on February 27, 2021. The trial was approved by the Harvard T.H. Chan School of Public Health Institutional Review Board (IRB13-0231), the Tanzanian National Health Research Ethics Sub-Committee (NIMR/HQ/R.8a/Vol.IX/1658), the Muhimbili University of Health and Allied Sciences Institutional Review Board (2016-05-25/AEC/Vol.X/01), and the Tanzania Food and Drugs Authority (TFDA13/CTR/0005/3). This study is reported as per the Consolidated Standards of Reporting Trials (CONSORT) guideline (S1 CONSORT Checklist).

### Participants

Pregnant women were eligible for enrollment in the trial if they (i) were adults ≥18 years of age, (ii) were pregnant and in the second trimester (12–27 weeks gestation) as determined by the reported date of last menstrual period at the time of randomization, (iii) were living with HIV, (iv) were receiving ART (new ART start or receiving ART before the current pregnancy), and (v) had a serum albumin-adjusted calcium level in the normal range (≤2.6 mmol/L) at the time of screening [23]. The trial excluded women who (i) did not intend to stay in the study area for 2 years after enrollment, (ii) were enrolled in any other clinical trial, or (iii) did not provide informed consent. Written informed consent in Kiswahili was obtained from all participants.

### Randomization, masking, and allocation concealment

Pregnant women were randomly assigned in a 1:1 ratio to either the vitamin D_3_ supplementation or placebo group, stratified by the study clinic. A non-study statistician computer-generated the randomization list with sequence blocks of 10 that were stratified by antenatal care clinic. The trial was triple-blind since the participants, study staff, investigators, and statisticians were masked to the randomized group assignments. The study statisticians who completed the primary analysis were blinded to group assignments by the use of coded labels. The vitamin D_3_ supplementation group received daily 3,000 IU vitamin D_3_ oral supplements (cholecalciferol) that were provided from randomization in the second trimester of pregnancy until trial discharge at 1 year postpartum. The US Institute of Medicine upper limit for vitamin D intake during pregnancy and lactation is 4,000 IU/day [24]. The placebo group received matching daily oral placebo supplements that were provided from randomization in pregnancy until trial discharge at 12 months postpartum. There was no difference between the vitamin D_3_ and placebo supplements in any aspect, including appearance, taste, smell, and weight. The vitamin D_3_ and placebo supplements were produced by Tishcon Corporation (Salisbury, MD, US). Complete allocation concealment was ensured through the use of regimen bottles that were prelabeled with sequentially numbered participant identification numbers. Eligible pregnant women who consented to trial randomization were assigned to the next available numeric participant identification number that corresponded to a set of prelabeled regimen bottles. Therefore, trial participants and research staff were not able to determine the allocated trial arm for any trial participant or identify trial participants who were on the same trial regimen.

### Procedures

The screening procedures for the trial were integrated into routine antenatal care. HIV testing was provided through the government antenatal care system at the antenatal care registration visit. Research nurses then identified pregnant women living with HIV and assessed eligibility criteria. Pregnant women living with HIV who met all eligibility criteria were asked to provide written informed consent for a blood draw to measure serum albumin and calcium concentrations to screen for hypercalcemia. Serum albumin and calcium concentrations at screening were assessed with the Roche Cobas Integra 400 Plus (Roche Diagnostics, Indianapolis, IN, US). Pregnant women in the normal range of albumin-adjusted calcium concentration (≤2.6 mmol/L) who returned to the study clinic for randomization when they were at 12–27 weeks gestation were eligible for enrollment.

At the randomization visit, all enrolled participants received a full clinical examination from a study physician, had their HIV disease stage assessed according to the WHO criteria, and were treated for concurrent morbidities per Tanzanian standard of care. Research nurses administered a standard questionnaire that collected information on sociodemographic characteristics, assessed participant-reported morbidities in the last 28 days, and measured height, weight, mid-upper arm circumference (MUAC), and blood pressure using standardized procedures. Follow-up study visits were conducted every 4 weeks during pregnancy, and starting at 32 weeks gestation all women received weekly phone calls and were reminded to call the study team at the time of labor. At all pregnancy study visits, study physicians performed a full clinical examination and assessed WHO HIV disease stage, and study nurses collected data on morbidities; assessed weight, MUAC, and blood pressure; and conducted a regimen pill count to assess adherence. The percent adherence was calculated by taking the total number of regimen pills taken by the participant from enrollment to delivery based on monthly pill counts divided by the total number of regimen pills the participant was expected to have consumed from enrollment to delivery based on 1 regimen pill per day. Study nurses/midwives attended labor and delivery to collect data on birthweight and other clinical outcomes. Mothers who delivered outside of Dar es Salaam were reached by phone to obtain data from clinical staff and medical records.

During the postnatal period, mother–infant pairs attended study visits at 6 weeks postpartum and every 4 weeks thereafter until trial discharge at 1 year postpartum. Women who experienced fetal death or had infants who died continued follow-up until 1 year postpartum. At all postpartum visits, mothers received a clinical examination and had WHO HIV disease stage assessed by study physicians, and nurses collected data on maternal morbidities; assessed weight, MUAC, and blood pressure; and conducted a regimen pill count. Infants received a full clinical examination from study physicians, and nurses collected data on infant morbidity history and breastfeeding practices and measured infant length and weight. Infant length was assessed in triplicate with standardized procedures using a rigid length board with an adjustable foot piece to 1-mm precision (SECA, Hamburg, Germany), and weight was measured in triplicate to the nearest 5 g using a digital scale (SECA, Hamburg, Germany). All infants received an HIV-1 DNA PCR test at 6 weeks and 12 months of age.

All mothers had blood collected at randomization, 32 weeks gestation, and 6 weeks, 6 months, and 12 months postpartum. Maternal serum albumin and calcium concentrations were assessed at each time point. All infants had blood collected at 6 weeks, 6 months, and 12 months of age. To assess the effect of maternal vitamin D_3_ supplementation on maternal and infant serum 25(OH)D concentrations, 320 mothers who had a baseline blood sample and at least 1 follow-up blood sample were randomly selected for serum 25(OH)D concentration assessment. All infants of the randomly selected mothers also had serum 25(OH)D assessed. Serum 25(OH)D concentrations were quantified at Boston Children’s Hospital with high-performance liquid chromatography tandem mass spectrometry (HPLC-MS/MS); day-to-day precision at various levels of 25(OH)D ranged from 5.6% to 8.5%.

### Outcomes

The primary efficacy outcomes were (i) maternal HIV progression or death from any cause, (ii) SGA live birth, and (iii) infant stunting at 1 year of age. Maternal HIV progression was defined as an increase in WHO HIV disease stage from that assessed at randomization [25]. SGA was defined as a birthweight less than the 10th percentile for gestational age by sex utilizing Oken standards [26]. Infant stunting at 12 months of age was defined by a length-for-age *z-*score (LAZ) < −2 on the WHO child growth standards [27]. Maternal hypercalcemia (serum albumin-adjusted calcium > 2.6 mmol/L) was evaluated as a targeted safety outcome [23].

Secondary outcomes were (i) maternal unsuppressed viral load, defined as an HIV-1 viral load > 1,000 copies/mL; (ii) fetal death (intrauterine death of a fetus at any time during pregnancy); (iii) miscarriage (fetal death at <28 weeks gestation); (iv) stillbirth (fetal death at ≥28 weeks gestation); (v) preterm birth (live birth at <37 weeks gestation); (vi) birthweight among live births; (vii) LBW (live birth birthweight < 2,500 g); (viii) mother-to-child transmission of HIV (infant HIV infection); (ix) infant mortality (death at ≤365 days); (x) infant LAZ, weight-for-age *z-*score (WAZ), and weight-for-length *z-*score (WLZ) at 1 year of age; (xi) infant wasting (WLZ < –2); (xii) infant underweight (WAZ < –2); (xiii) infant LAZ, WAZ, and WLZ trajectory in the first year of life; and (xiv) maternal and infant serum 25(OH)D concentrations. Early during the conduct of the trial, the HIV care and treatment program discontinued routine CD4 T-cell assessment after ART initiation, and therefore we do not present treatment effects on this secondary outcome included in the protocol. As a sensitivity analysis, we assessed the effect of vitamin D_3_ on SGA birth as defined by INTERGROWTH-21st standards, which were published during the conduct of the trial [28]. We also analyzed death of the mother, mean gestation duration, and neonatal mortality (death at ≤28 days) as post hoc outcomes. These 3 post hoc outcomes were not predefined as secondary outcomes in the study protocol.

### Statistical analysis

The full details of the trial sample size calculation for the 3 primary outcomes (maternal HIV progression or death, SGA birth, and infant stunting) can be found in the published protocol [22]. Briefly, the trial target sample size was 2,300 pregnant women living with HIV based on power calculations that assumed 1:1 randomization to vitamin D_3_ or placebo and a nominal type I error rate (alpha) of 0.05 for each primary outcome. We did not account for multiple comparisons. For maternal HIV progression of death, we assumed 90% retention of pregnant women until trial discharge, and therefore the trial had >80% power to detect a relative risk (RR) of 0.70 for maternal HIV progression or death if the event rate was ≥15% in the placebo group. For SGA birth, we assumed that 90% of pregnancies would result in live births with birthweight data available, and therefore we had >90% power to detect a relative risk of 0.75 for SGA birth if the cumulative incidence was ≥14% in the placebo group. For infant stunting, we assumed that 85% of liveborn infants would be living and would have length assessed at 1 year of age, and therefore we had >90% power to detect a relative risk of 0.80 for infant stunting if the cumulative incidence of infant stunting was ≥20% in the placebo group.

An intent-to-treat analysis with a complete case approach was used as the primary analytic strategy for all analyses (S1 Analysis Plan). The log-rank test was used to evaluate the effect of vitamin D_3_ supplementation on the incidence of maternal HIV progression or death from any cause, and Cox proportional hazards models were used to estimate hazard ratios (HRs). Analyses of SGA birth, infant stunting, and other binomial infant outcomes were conducted with generalized estimating equations (GEEs) including a compound symmetry working correlation matrix to account for correlation due to multiple gestation (twins) and a log link and binomial variance function to produce relative risks. Secondary continuous non-repeated outcomes, including birthweight and infant LAZ, WLZ, and WAZ at 1 year of age, were evaluated with generalized linear mixed-effects models with a random intercept, a compound symmetric covariance structure, and sandwich variance estimators. For secondary continuous outcomes with repeated measurements—including infant LAZ, WLZ, and WAZ trajectories from 6 weeks to 1 year of age and maternal and infant 25(OH)D concentration trajectories—generalized linear mixed-effects models with a random intercept, a compound symmetric covariance structure, and sandwich variance estimators were used to assess the effect of vitamin D_3_ supplementation on the longitudinal continuous outcome. The overall statistical significance of the difference in repeated measures over time between randomized groups was determined from an interaction term between randomized regimen and time. All analyses accounted for the study clinic with fixed effects due to stratified randomization.

We conducted several sensitivity analyses for the primary outcomes. To examine the potential for baseline imbalance to affect our results, we conducted sensitivity analyses for the primary outcomes adjusting for the following characteristics at baseline (randomization): maternal age, gestational age, maternal BMI, wealth quintile, WHO HIV disease stage, CD4 T-cell count, and timing of ART initiation. We conducted a post hoc analysis of maternal HIV progression and death of the mother, separately. We conducted sensitivity analyses for birth outcomes and infant growth outcomes restricted to singleton births. Further, we assessed the potential for bias due to dependent censoring in the infant stunting analysis (missing length data) by conducting a sensitivity analysis that used inverse probability of censoring weighting (IPCW) [29]. Stabilized censoring weights were constructed in models that included maternal age, gestational age at randomization, maternal BMI, household wealth quintile, WHO HIV disease stage, CD4 T-cell count, timing of ART initiation, and infant sex, birthweight, and gestational age at birth.

In exploratory analyses, we examined the potential for modification of the effect of maternal vitamin D supplementation on the primary outcomes by predefined baseline maternal factors including maternal age, gestational age at randomization, maternal body mass index, socioeconomic status, WHO HIV disease stage, ART initiation before or after conception, and trial regimen adherence [22]. We used the Wald test for risk ratio homogeneity to assess the statistical significance of effect modification. All statistical analyses were done with SAS, version 9.3.

## Results

**Fig 1:**
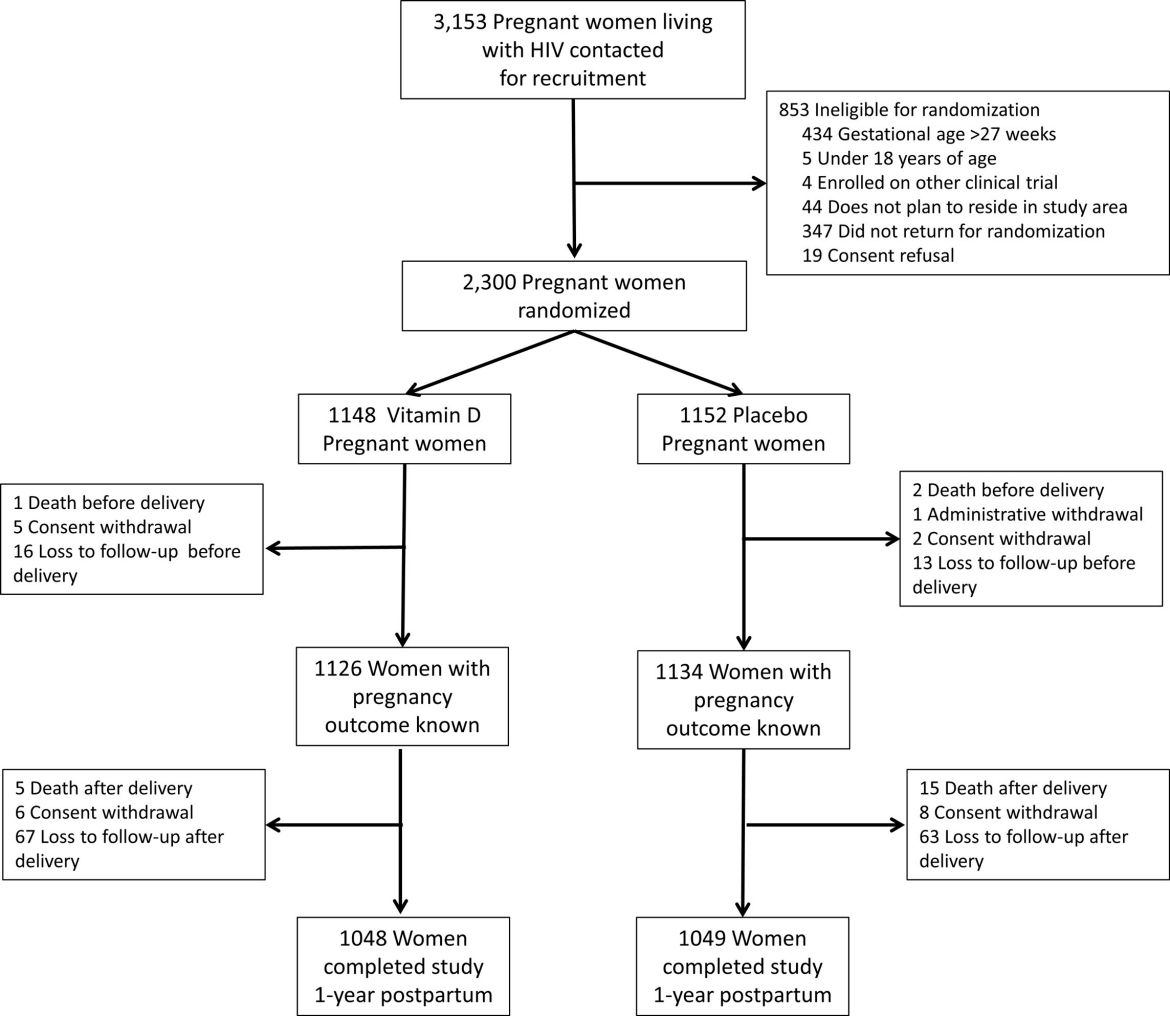
Trial flow diagram for mothers.

**Fig 2:**
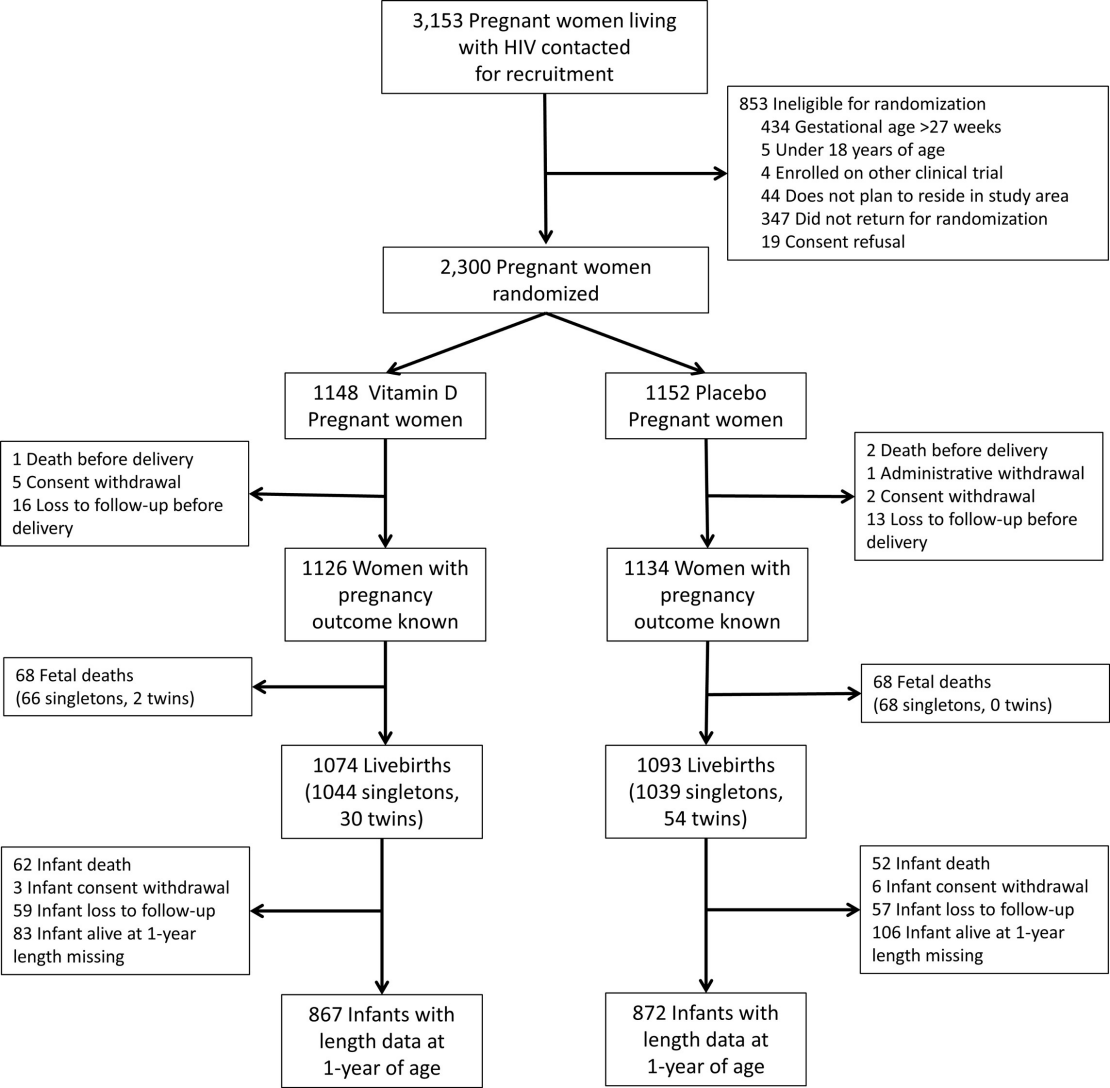
Trial flow diagram for infants.

The trial flow diagrams for mothers and infants are presented in Figs 1 and 2, respectively. A total of 3,153 pregnant women living with HIV were screened for recruitment, of whom 2,300 were randomized (1,148 to vitamin D_3_ and 1,152 to placebo). One pregnant woman in the placebo group was administratively withdrawn after she tested negative on an HIV-1 ELISA at the time of labor and subsequently on multiple ELISA and PCR tests after stopping ART; the participant was excluded from all outcome analyses. Birth outcome data were available for 1,126 women (98.3%) in the vitamin D_3_ group and 1,134 women (98.4%) in the placebo group. There were a total of 1,074 live births in the vitamin D_3_ group and 1,093 live births in the placebo group. At 1 year of age, vital status was known for 1,012 infants (94.2%) in the vitamin D_3_ group and 1,030 infants (94.2%) in the placebo group. The proportion of mothers lost to follow-up at 1 year postpartum was similar between the randomized groups: 6.6% in the vitamin D_3_ group (83 of 1,148 women randomized) and 6.6% in the placebo group (76 of 1,152 women randomized) (*p*-value for difference between groups: 0.71). The proportion of children lost to follow-up at 1 year of age (unknown vital status) was 5.5% in the vitamin D_3_ group (59 of 1,074 live births) and 5.2% in the placebo group (57 of 1,093 live births) (*p*-value for difference between groups: 0.77). Data on infant length at 1 year of age was available for 867 infants (85.7% of live births excluding deaths) in the vitamin D_3_ group and 872 infants (83.8% of live births excluding deaths) in the placebo group, and there was no difference in missingness between the randomized groups (*p*-value for difference between groups: 0.15).

Baseline characteristics were similar between the randomized groups (Table 1). Maternal adherence to the trial regimen was similarly high between treatment groups; the median (interquartile range) adherence was 88.2% (63.0%, 97.8%) in the vitamin D group and 85.5% (60.5%, 96.0%) in the placebo group.

**Table 1:** Baseline characteristics of trial population of pregnant women living with HIV stratified by randomization group.

Characteristic	Vitamin D_3_ (*N =* 1,148), *n* (%)	Placebo (*N =* 1,152), *n* (%)
Age		
18–24 years	180 (15.7)	189 (16.4)
25–34 years	673 (58.6)	666 (57.8)
35+ years	295 (25.7)	297 (25.8)
Maternal education		
No formal education	118 (10.3)	132 (11.5)
Primary	675 (58.8)	669 (58.1)
Secondary/advanced	354 (30.8)	350 (30.4)
Missing	1 (0.1)	1 (0.1)
Weeks gestation at randomization		
12–16.9 weeks	251 (21.9)	235 (20.4)
17–22.9 weeks	538 (46.9)	549 (47.7)
23–27 weeks	359 (31.3)	368 (31.9)
Body mass index at randomization		
<18.5 kg/m^2^	24 (2.1)	40 (3.5)
18.5–24.9 kg/m^2^	522 (45.5)	519 (45.1)
25.0–29.9 kg/m^2^	370 (32.2)	382 (33.2)
≥30.0 kg/m^2^	223 (19.4)	202 (17.5)
Missing	9 (0.8)	9 (0.8)
WHO HIV disease stage		
I	1,004 (87.5)	970 (84.2)
II	64 (5.6)	78 (6.8)
III	73 (6.4)	93 (8.1)
IV	7 (0.6)	11 (1.0)
CD4 T-cell count, cells/μL		
<200	100 (8.7)	96 (8.3)
200–349	139 (12.1)	139 (12.1)
350–499	156 (13.6)	155 (13.5)
≥500	149 (13.0)	148 (12.9)
Missing	604 (52.6)	614 (53.3)
Timing of ART initiation		
During this pregnancy	689 (60.0)	675 (58.6)
Before conception	459 (40.0)	477 (41.4)
ART regimen		
Tenofovir/lamivudine/efavirenz	1,137 (99.0)	1,136 (98.8)
Other	11 (1.0)	16 (1.4)

ART, antiretroviral therapy; HIV, human immunodeficiency virus; WHO, World Health Organization.

### Maternal HIV progression and death

During follow-up, 307 (13.3% of participants) maternal HIV progression or death events were recorded. There was no difference in the hazard of maternal HIV progression or death between the vitamin D_3_ and placebo groups, with 166 events during 1,461 person-years of follow-up in the vitamin D_3_ group and 141 events during 1,469 person-years of follow-up in the placebo group (HR 1.21, 95% CI 0.97 to 1.52, *p =* 0.09; Table 2). There was no difference in the findings of the effect of vitamin D_3_ on maternal HIV progression or death in a sensitivity analysis that included covariates for baseline variables to account for potential baseline imbalance between the randomized groups (adjusted HR 1.17, 95% CI 0.93 to 1.46, *p =* 0.18). There was no evidence of effect modification for any baseline maternal factors, except maternal BMI at randomization, where the effect of vitamin D_3_ on maternal HIV progression or death appeared more negative for women with a baseline BMI < 25 kg/m^2^ as compared to those with a baseline BMI ≥ 25.0 kg/m^2^ (Table A in S1 Appendix). In a post hoc analysis of the composite endpoint, we examined the effect of maternal vitamin D_3_ supplementation on death and HIV progression separately (Table 2). The hazard of death was lower in the vitamin D_3_ group than in the placebo group (HR 0.35, 95% CI 0.14 to 0.89, *p =* 0.02); 6 women died in the vitamin D_3_ group, and 17 women died in the placebo group. Causes of death for the women by treatment group are presented in Table B in S1 Appendix. In contrast, the hazard of HIV progression (defined by any upgrade in WHO HIV disease stage) was greater in the vitamin D_3_ group than in the placebo group (HR 1.32, 95% CI 1.04 to 1.66, *p =* 0.02). There was no effect of vitamin D_3_ supplementation on the risk of women having an unsuppressed HIV viral load at the first viral load test post-randomization (Table 2).

**Table 2:** Effect of vitamin D_3_ supplementation on maternal HIV progression or death.

Outcome	Vitamin D_3_, *n/N* (%)	Placebo, *n/N* (%)	Hazard ratio (95% CI)	*p*-Value^1^
Maternal HIV progression^2^ or death (primary outcome)	166/1,148 (14.5%)	141/1,151 (12.3%)	1.21 (0.97, 1.52)	0.09
Maternal death	6/1,148 (0.5%)	17/1,151 (1.5%)	0.35 (0.14, 0.89)	0.02
Maternal HIV progression excluding death	161/1,148 (14.0%)	126/1,151 (11.0%)	1.32 (1.04, 1.66)	0.02
Maternal unsuppressed HIV-1 viral load (>1,000 copies/mL)^3^	69/794 (8.7%)	71/777 (9.1%)	0.96 (0.70, 1.31)	0.79

^1^Log-rank test *p*-value for maternal HIV progression or death, maternal death, and maternal HIV progression excluding death.

^2^Any upgrade in WHO HIV disease stage.

^3^Median (interquartile range) timing of viral load assessment post-randomization: 216 days (146, 345).

CI, confidence interval; HIV, human immunodeficiency virus; WHO, World Health Organization.

### SGA births and secondary birth outcomes

Table 3 presents the effect of maternal vitamin D_3_ supplementation on SGA births and secondary birth outcomes. There was no difference in the risk of the primary outcome of SGA live birth, defined by the Oken standard, between the vitamin D_3_ and placebo groups (RR 1.03, 95% CI 0.87 to 1.22, *p =* 0.70). The findings were similar when restricted to singletons (Table C in S1 Appendix) and in a sensitivity analysis adjusting for baseline maternal factors (RR 1.06, 95% CI 0.90 to 1.25, *p* = 0.50). There was no evidence of effect modification by baseline maternal factors (Table D in S1 Appendix). There was also no effect on SGA birth defined by the INTERGROWTH-21st standards in a sensitivity analysis (Table 3). There was also no difference in the risk of fetal death, miscarriage, stillbirth, LBW, or mean birthweight between the vitamin D_3_ and placebo groups (Table 3). The risk of preterm birth (<37 weeks gestation) was greater in the vitamin D_3_ group than in the placebo group (RR 1.17, 95% CI 1.00 to 1.36, *p =* 0.04). In a sensitivity analysis restricted to singletons, the relative risk of preterm was similar but did not reach statistical significance (RR 1.15, 95% CI 0.98 to 1.34, *p =* 0.08; Table C in S1 Appendix). In a post hoc analysis, mean gestation duration was significantly shorter in the vitamin D_3_ group than in the placebo group (mean difference −0.36 weeks, 95% CI −0.64 to −0.09, *p =* 0.01; Table 3), and the results were robust to restriction of the analysis to singletons (Table C in S1 Appendix).

**Table 3:** Effect of maternal vitamin D_3_ supplementation on small-for-gestational-age live births and secondary birth and infant outcomes.

Outcome	Vitamin D_3_, number of events/number at risk (%) or mean (SD)	Placebo, number of events/number at risk (%) or mean (SD)	Relative risk (95% CI) or mean difference	*p-*Value
Fetal death	68/1,142 (6.0%)	68/1,161 (5.9%)	1.02 (0.73, 1.40)	0.93
Miscarriage (<28 weeks gestation)	23/1,142 (2.0%)	18/1,161 (1.6%)	1.30 (0.70, 2.39)	0.40
Stillbirth (≥28 weeks gestation)	45/1,119 (4.0%)	50/1,143 (4.4%)	0.92 (0.62, 1.36)	0.67
*Among live births*				
Birthweight (g)	3,086 (577)	3,080 (518)	−2 (−49, 46)	0.95
Low birthweight (<2,500 g)	111/1,070 (10.4%)	106/1,091 (9.7%)	1.15 (0.88, 1.50)	0.32
Duration of gestation (weeks)	38.6 (3.3)	38.9 (3.0)	−0.36 (−0.64, −0.09)	0.01
Preterm birth (<37 completed weeks gestation)	281/1,074 (26.2%)	242/1,093 (22.1%)	1.17 (1.00, 1.36)	0.04
Small-for-gestational-age birth—Oken standard (<10th percentile) (primary outcome)	229/1,070 (21.4%)	236/1,091 (21.6%)	1.03 (0.87, 1.22)	0.70
Small-for-gestational-age birth—INTERGROWTH-21st standard (<10th percentile)	198/1,070 (18.5%)	223/1,091 (20.4%)	0.94 (0.79, 1.12)	0.49
Infant HIV infection	22/1,074 (2.1%)	23/1,093 (2.1%)	0.97 (0.54, 1.71)	0.90
Neonatal mortality (≤28 days)	34/1,074 (3.2%)	23/1,093 (2.1%)	1.49 (0.88, 2.52)	0.14
Infant mortality (≤365 days)	62/1,074 (5.8%)	52/1,093 (4.8%)	1.20 (0.84, 1.72)	0.32

CI, confidence interval; HIV, human immunodeficiency virus; SD, standard deviation.

### Infant HIV infection and death

During follow-up, a total of 45 infants (2.1%) were diagnosed with HIV infection; there was no difference in the risk of mother-to-child HIV transmission between the treatment groups (Table 3). There were 114 infant deaths (5.3%) recorded, and there was no difference between the vitamin D_3_ and placebo groups (Table 3). Causes of infant death by treatment group are presented in Table E in S1 Appendix. There was no difference in the findings for HIV infection or infant death when restricting the analyses to singletons (Table C in S1 Appendix).

### Infant stunting and anthropometric measures

The effects of maternal vitamin D_3_ supplementation on infant stunting and secondary infant growth outcomes at 1 year of age are presented in Table 4. There was no effect of maternal vitamin D_3_ supplementation on the risk of infant stunting (RR 1.00, 95% CI 0.92 to 1.10, *p =* 0.95). The findings were similar when restricting the analysis to singletons (Table F in S1 Appendix) and in a sensitivity analysis adjusting for maternal baseline factors (RR 1.00, 95% CI 0.90 to 1.10, *p =* 0.98). A sensitivity analysis using IPCW to account for missing length data also found similar results (RR 1.02, 95% CI 0.93 to 1.12, *p =* 0.73). There was no evidence of effect modification by baseline maternal factors (Table G in S1 Appendix). There was also no difference in the secondary growth outcomes of LAZ, WLZ, wasting, WAZ, and underweight at 1 year of age between the treatment groups (Table 4). There was no difference in infant LAZ, WLZ, and WAZ trajectories from 6 weeks to 1 year of age by treatment group (Figs A–C in S1 Appendix).

**Table 4:** Effect of maternal vitamin D_3_ supplementation on infant stunting and other anthropometric measures at 1 year of age.

Infant growth outcome at 12 months of age	Vitamin D_3_, number of events/number at risk (%) or mean (SD)	Placebo, number of events/number at risk (%) or mean (SD)	Relative risk (95% CI) or mean difference	*p*-Value
LAZ	−1.87 ± 1.46	−1.82 ± 1.45	−0.04 (−0.18, 0.09)	0.52
Stunting (LAZ < −2) (primary outcome)	407/867 (46.9%)	413/872 (47.4%)	1.00 (0.92, 1.10)	0.95
WLZ	0.68 ± 1.38	0.77 ± 1.39	−0.09 (−0.23, 0.05)	0.21
Wasting (WLZ < −2)	18/757 (2.4%)	14/749 (1.9%)	1.27 (0.63, 2.55)	0.50
WAZ	−0.44 ± 1.17	−0.34 ± 1.19	−0.10 (−0.23, 0.02)	0.10
Underweight (WAZ < −2)	69/767 (9.0%)	55/758 (7.3%)	1.24 (0.88, 1.74)	0.21

CI, confidence interval; LAZ, length-for-age *z-*score; SD, standard deviation; WAZ, weight-for-age *z-*score; WLZ, weight-for-length *z-*score.

### Maternal and infant 25(OH)D concentrations

**Fig 3:**
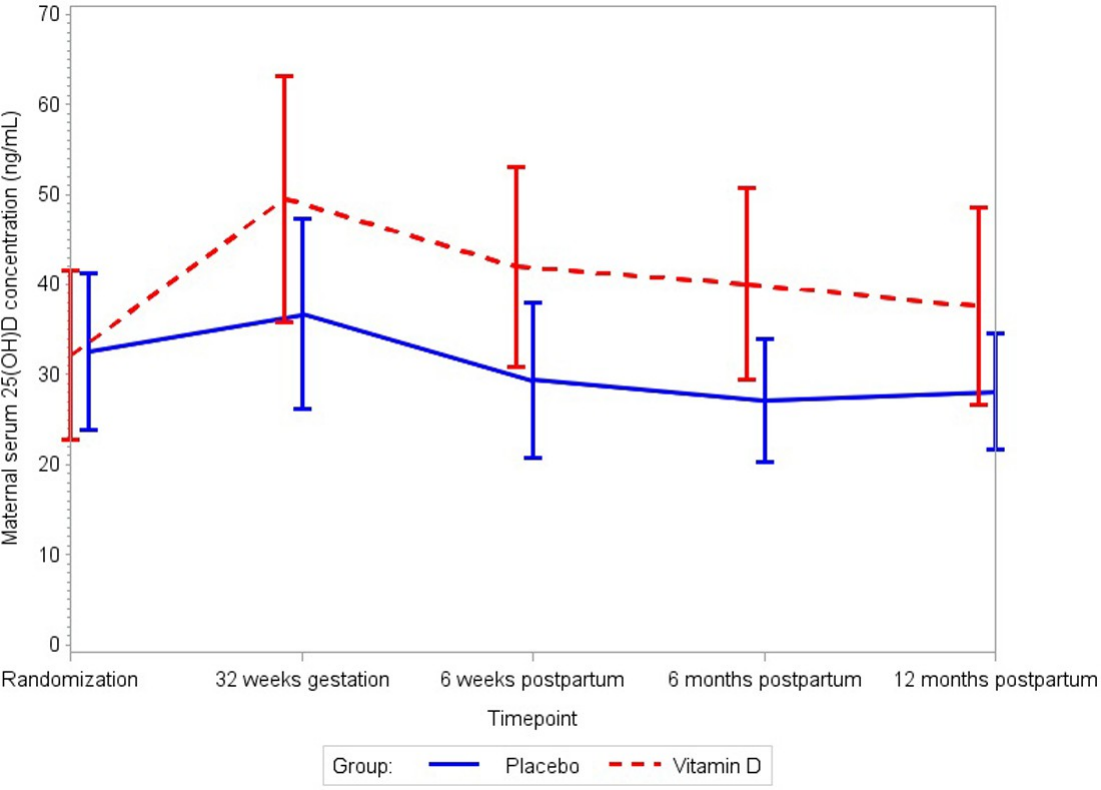
Effect of maternal vitamin D_3_ supplementation on mean maternal serum 25(OH)D concentrations. Whiskers indicate standard deviation. *p-*Value for difference in trajectory for maternal serum 25(OH)D concentration < 0.001. 25(OH)D, 25-hydroxyvitamin D.

**Fig 4:**
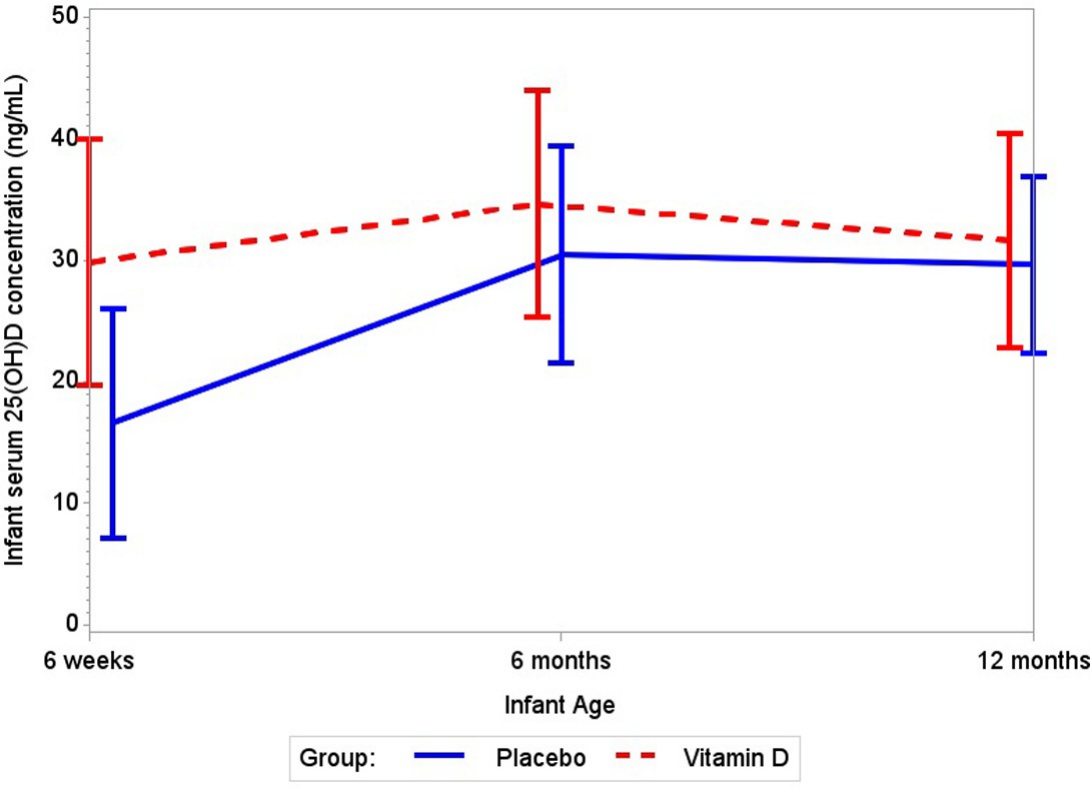
Effect of maternal vitamin D_3_ supplementation on mean infant serum 25(OH)D concentrations. Whiskers indicate standard deviation. *p-*Value for difference in trajectory for infant serum 25(OH)D concentration < 0.001. 25(OH)D, 25-hydroxyvitamin D.

The effects of maternal vitamin D_3_ supplementation on maternal and infant serum 25(OH)D concentrations are presented in Figs 3 and 4, respectively. Maternal vitamin D_3_ supplementation increased maternal 25(OH)D concentrations during follow-up as compared to placebo (*p*-value for difference in trajectory between groups: <0.001). Maternal 25(OH)D concentrations were higher in the vitamin D_3_ group at all post-randomization timepoints including 32 weeks gestation and 6 weeks, 6 months, and 12 months postpartum (Table H in S1 Appendix). The mean difference in maternal 25(OH)D between the vitamin D_3_ and placebo groups was 12.8 ng/mL (95% CI 9.4 to 16.1, *p <* 0.001) at 32 weeks gestation, 12.6 ng/mL (95% CI 10.0 to 15.2, *p <* 0.001) at 6 weeks postpartum, 12.9 ng/mL (95% CI 10.3 to 15.5, *p <* 0.001) at 6 months postpartum, and 9.5 ng/mL (95% CI 7.0 to 12.0, *p <* 0.001) at 12 months postpartum. Infant 25(OH)D concentrations during follow-up significantly differed between the vitamin D_3_ and placebo groups (*p*-value for difference in trajectory: <0.001). Infant serum 25(OH)D concentrations were higher in the vitamin D_3_ group at 6 weeks and 6 months of age, but there was no difference between the treatment groups at 12 months of age (Table I in S1 Appendix). The mean difference in infant 25(OH)D between the vitamin D_3_ and placebo groups was 13.2 ng/mL (95% CI 10.6 to 15.9, *p <* 0.001) at 6 weeks, 4.2 ng/mL (95% CI 1.6 to 6.8, *p =* 0.002) at 6 months, and 2.0 ng/mL (95% CI −0.4 to 4.4, *p =* 0.10) at 12 months of age.

### Maternal hypercalcemia and safety outcomes

No maternal hypercalcemia events were recorded. There was 1 hypersensitivity reaction to the trial supplements for a pregnant woman in the placebo group.

## Discussion

Maternal vitamin D_3_ supplementation during pregnancy and lactation did not significantly affect the risk of maternal HIV disease progression or death, SGA live births, or infant stunting at 1 year of age. There was also no effect of vitamin D_3_ supplementation on most secondary maternal and infant outcomes. However, we observed that the risk of maternal death was lower, but the risks of HIV progression and preterm birth were greater, for mothers in the vitamin D_3_ group as compared to the placebo group. The vitamin D_3_ supplementation regimen increased maternal and infant serum 25(OH)D concentrations and did not increase the risk of maternal hypercalcemia.

Our finding of no effect of vitamin D_3_ supplementation on the composite primary outcome of maternal HIV progression or death is not aligned with observational evidence from cohort studies, including those conducted in Tanzania, that have found that vitamin D deficiency is associated with increased risk of HIV progression and mortality for people living with HIV [12,13]. Nevertheless, the null findings are generally consistent with a recent randomized controlled trial of vitamin D_3_ supplementation conducted among HIV-infected adult men and non-pregnant women initiating ART in Tanzania that found no effect on the risk of death during the first year of treatment [30]. It is possible that 25(OH)D concentrations and vitamin D status as determined in observational studies may be a marker of immune activation, comorbidities, or other confounding factors rather than indicating that vitamin D has a causal relationship with HIV progression and death. Further, while in vitro studies suggest that vitamin D may reduce HIV-1 replication [31–33], we found no effect of vitamin D supplementation on HIV-1 viral suppression, an important contributor to the risk of HIV progression and death for individuals on ART.

In a post hoc analysis separating the composite endpoint HIV progression or death, we observed that women in the vitamin D_3_ group had a lower risk of death during follow-up, but increased risk of HIV progression, as compared to women in the placebo group. Foremost, it is important to note these post hoc findings may be due to chance, particularly given the small number of deaths and some degree of competing risks. These opposing findings are difficult to explain and require additional research, but we hypothesize that the findings may be attributable to the range of effects of vitamin D and the complexity of the state of pregnancy in the context of HIV and ART [34]. Immunologic pathways should be evaluated as pregnancy is characterized by Th2 response predominance and downregulation of Th1 responses, which result in immune tolerance, and this immunosuppressive state is also associated with increased susceptibility to intracellular pathogens [34,35]. Vitamin D also downregulates Th1 responses and therefore may further shift the Th1/Th2 balance towards increased risk of infections [36]. Indeed, we found that the vitamin D_3_ group had an increased risk of HIV progression as defined by increased WHO HIV disease stage, which is primarily defined by new-onset opportunistic infections. Further, untreated progression of HIV infection to AIDS is associated with a shift from Th1 to Th2 responses, while ART reverses the shift to a more balanced response [37,38]. Therefore, vitamin D_3_ supplementation may counterbalance the effects of ART. Nevertheless, vitamin D has multiple other immunomodulatory effects that may simultaneously reduce the risk of mortality from infectious diseases, as well as physiological effects that may reduce the risk of other causes of death [36,39,40]. Additional research is needed to untangle the potentially complex effects of vitamin D_3_ supplementation on immune responses and clinical outcomes in the context of pregnancy, HIV, and ART.

We found no effect of maternal vitamin D_3_ supplementation on the risk of SGA live birth, an indicator of fetal growth restriction. There have been multiple proposed mechanisms by which vitamin D in pregnancy may affect fetal growth, including improved immune function, placental function, or fetal skeletal development as well as reduced risk of preeclampsia [41–43]. Randomized trials have found mixed effects of vitamin D supplementation on SGA births. A meta-analysis of 5 small randomized trials of vitamin D supplementation that included a total of 793 live births found an approximately 30% reduction in the risk of SGA birth [44]; however, the subsequently published Maternal Vitamin D for Infant Growth (MDIG) trial conducted in Bangladesh found no effect of prenatal maternal vitamin D supplementation on SGA birth among 834 live births, which is consistent with our findings [45].

We also found that maternal vitamin D_3_ supplementation did not affect birthweight or risk of LBW, but we observed that the vitamin D_3_ group had increased risk of preterm birth and decreased mean gestation duration, by approximately 2.5 days, compared to the placebo group. It is important to consider that effects on preterm birth and gestation duration may be attributable to chance. The most recent Cochrane review found no effect of maternal vitamin D supplementation on the risk of preterm birth in 7 randomized trials that included 1,640 pregnant women, but there was a 45% reduction in the risk of LBW in 5 trials that included a total of 697 women [20]. However, the recent MDIG trial found no effect of multiple prenatal vitamin D_3_ regimens on birthweight, LBW, or preterm birth [45]. Our findings of a negative effect of vitamin D supplementation on preterm birth and duration of gestation were unexpected given the relatively consistent epidemiological literature indicating that vitamin D deficiency is associated with an increased risk of preterm birth [34]. Preterm birth is associated with a pro-inflammatory Th1 profile, and therefore the anti-inflammatory actions of vitamin D would be expected to reduce the risk [34]. However, we found that mothers in the vitamin D_3_ group had increased risk of HIV progression, which is a risk factor for preterm birth and may represent a potential mechanism that contributed to our findings [46].

We also found no effect of vitamin D supplementation during pregnancy and lactation on infant stunting or other measures of infant growth. Small trials in Bangladesh and the UK found beneficial effects of maternal vitamin D supplementation on infant linear growth [47,48]. However, these findings were not replicated in the MDIG trial in Bangladesh, which found no effect of various prenatal or postpartum vitamin D_3_ supplementation regimens on infant linear growth [45]. Two of the potential pathways for maternal vitamin D supplementation during pregnancy and lactation to affect infant growth are through improved birth outcomes and improved infant vitamin D status. While there was no evidence of benefit in terms of birth outcomes, maternal vitamin D supplementation increased infant serum 25(OH)D concentrations. The improvements in infant vitamin D status at 6 weeks and 6 months in our trial are likely attributable to improved vitamin D content in breastmilk during the period of exclusive breastfeeding [49]; however, the frequency and intensity of breastfeeding tend to decrease during the period of complementary feeding in the second half of infancy, possibly contributing to there being no effect on infant 25(OH)D concentration at 12 months of age. Nevertheless, the improvements in infant vitamin D status did not translate into effects on child growth outcomes.

Our trial has several limitations. First, we enrolled pregnant women in the second trimester of pregnancy, and therefore additional research on vitamin D supplementation initiated preconceptionally or in the first trimester of pregnancy is needed. Second, we calculated gestational age based on maternal report of last menstrual period, which likely led to some degree of mismeasurement of the duration of gestation and misclassification of prematurity; however, this misclassification was likely non-differential between treatment arms and therefore would attenuate measures of effect on preterm birth and gestation duration. Third, we did not detect any clinically diagnosed cases of infant rickets, but we did not perform biochemical or other enhanced screening measures that may have led to the identification of cases. In addition, we analyzed multiple secondary and post hoc outcomes, and therefore we cannot rule out that the findings on maternal death, gestation duration, and preterm birth were due to type I errors (incorrectly rejecting the null) attributable to multiple testing. Last, our trial was conducted among women living with HIV in Tanzania, and therefore the findings may not be generalizable to HIV-uninfected women or those living in different contexts, particularly where severe vitamin D deficiency is prevalent. However, it is important to note that there was no effect of vitamin D_3_ supplementation on birth outcomes or child growth in the MDIG trial in Bangladesh, where at baseline approximately 60% of the pregnant women had 25(OH)D concentrations of <12 ng/mL.

We found no effect of maternal vitamin D_3_ supplementation during pregnancy and lactation on the risk of maternal HIV progression or death, SGA live birth, or infant stunting at 1 year of age. Surprisingly, we observed a beneficial effect of vitamin D_3_ supplementation on the risk of death for mothers, but negative effects on maternal HIV progression, duration of gestation, and risk of prematurity. However, these secondary and post hoc findings may be due to chance, should be interpreted with caution, and require replication before one assumes beneficial effects or safety risks. Taken together, our trial findings do not support policy change to implement routine vitamin D supplementation for pregnant and lactating women living with HIV in Tanzania and similar contexts.

## Supporting information

S1 CONSORT Checklist Checklist of information to include when reporting a randomized controlled trial. (DOC)

S1 Protocol Trial of vitamin D in HIV progression, birth outcomes, and child health—study protocol. (DOCX)

S1 Analysis Plan Trial of vitamin D in HIV progression, birth outcomes, and child health—analysis plan. (DOCX)

S1 Appendix Supplementary tables and figures. (DOCX)

## Abbreviations

25(OH)D: 25-hydroxyvitamin D
ART: antiretroviral therapy
HEU: HIV-exposed uninfected
HR: hazard ratio
IPCW: inverse probability of censoring weighting
LAZ: length-for-age *z-*score
LBW: low birthweight
MUAC: mid-upper arm circumference
RR: relative risk
SGA: small-for-gestational-age
WAZ: weight-for-age *z-*score
WLZ: weight-for-length *z-*score
