# Supplementary material for: Vitamin D3 supplementation during pregnancy and lactation for women living with HIV in Tanzania: A randomized controlled trial
Source: PLoS Med. 2022 Apr 15;19(4):e1003973. doi: 10.1371/journal.pmed.1003973 (PMC9012360; doi:10.1371/journal.pmed.1003973)
Supplement: S1 Protocol — (DOCX) [file pmed.1003973.s002.docx]

**TITLE: Trial of Vitamin D in HIV Progression, Birth Outcomes, and Child Health (TOV5)**

**Study Protocol**

**Version 2.0 / February 16, 2015**

**0.1 SUMMARY ABSTRACT**

**Background:** The overall project goal is to investigate maternal vitamin D3 (cholecalciferol) supplementation starting during the second trimester of pregnancy and continued until 12 months post-partum as a simple and low cost intervention to prolong and improve quality of life for HIV-infected pregnant women and their children in Tanzania and similar resource limited settings. This will be done through a randomized, double-blind, placebo-controlled clinical trial.

**Rationale:** HIV-infected pregnant women receiving antiretroviral therapy in Tanzania and many resource-limited settings experience high rates of mortality, morbidity, and adverse birth outcomes and their children also exhibit poor linear growth. This randomized trial may provide evidence that maternal vitamin D3 supplements prolong and improve the quality of life for HIV-infected pregnant women and their children, which may result in adding these supplements to standard PMTCT care.

**Objectives:** The overall objective is to conduct a randomized, double-blind, placebo-controlled trial to determine the effect of maternal vitamin D3 oral supplements containing 3,000 IU taken from the second trimester of pregnancy until 12 months post-partum on (a) maternal HIV progression or death, (b) small-for- gestational age infants, and (c) infant stunting at 1 year of age.

**Methods:** Briefly, we will enroll 2,300 HIV-infected pregnant women at 12-27 weeks gestation (second trimester) who are enrolled in the Management and Development for Health (MDH) President's Emergency Plan for AIDS Relief (PEPFAR) program in Dar-es-Salaam, Tanzania. HIV-infected mothers who consent for enrollment in the trial will be randomized to receive an oral supplement regimen of one of two groups: (a) vitamin D3 oral supplements containing 3,000 IU taken daily from the second trimester of pregnancy until 12 months post-partum or (b) placebo supplements taken daily from the second trimester of pregnancy until 12 months postpartum. No infants will receive supplements. HIV-infected women and children will be followed at regular study visits and during labor and delivery to assess efficacy and safety endpoints.

**Study clinics:** Mnazi Mmoja (AKC), Buguruni, Mbagala Rangi Tatu, Magomeni, Tandale, Mbagala Round Table and Sinza PMTCT clinics.

**1.0 INTRODUCTION AND LITERATURE REVIEW**

**1.1 ART in Pregnancy and Option B+:** Antiretrovirals are the principal tool utilized to prevent mother-to-child HIV transmission (PMTCT) worldwide, primarily owing to their ability to reduce maternal viral load during antenatal, delivery, and lactation periods [1]. Since the early 2000’s, well-resourced countries in North America and Europe have been able to reduce the risk of mother-to-child transmission (MTCT) of HIV infection to 1-2% largely through use of three-drug combination ART [2,3]. In contrast, during this period PMTCT programs in resource-limited settings widely relied on simple monotherapy regimens due to high costs of complex regimens, lack of health infrastructure, and high HIV burden [4]. After investments in infrastructure and development of better regimens, the World Health Organization revised guidelines for treatment of HIV-infected pregnant women in 2010. The WHO 2010 guidelines recommended two approaches for PMTCT prophylaxis: Option A and Option B [5]. Specifically in Option B, pregnant women initiate three drug ART prophylaxis during the antenatal period and continue until the cessation of breastfeeding and their infants receive daily nevirapine (NVP) or zidovudine (ZDV) prophylaxis until 6 weeks. In 2011 the Malawian government introduced an altered version of Option B termed Option B+, primarily to sidestep the difficulties of CD4 T-cell testing [6]. In Option B+, all HIV-infected pregnant women initiate lifelong triple-drug ART, irrespective of clinical or immunologic status, and infants receive NVP until 6 weeks. In addition to avoiding a CD4 based criteria, the rationale for utilizing Option B+ is that pregnant women take triple antiretrovirals for their own health as well as to prevent MTCT for the current as well as future pregnancies and mothers avoid the risks of starting and stopping antiretrovirals. **Based on the Malawian experience, the Tanzania PMTCT standard of care and Management and Development for Health (MDH) PEPFAR program commenced Option B+ in Dar-es-Salaam in 2013.**

**1.2 Maternal ART and Birth Outcomes:** It has long been recognized that untreated HIV infection during pregnancy significantly increases the risk of most adverse birth outcomes (spontaneous abortion, fetal abnormality, intrauterine growth retardation, preterm delivery, etc.); however, the effect of ART during pregnancy on birth outcomes remains unclear. There have been some recent studies and reports that suggest use of ART during pregnancy may increase risk of premature delivery and small-for-gestational age (SGA) infants, while others have found no association [7-14]. A study by Chen et al. in Botswana determined HIV- infected pregnant women who received ART before pregnancy had higher odds of preterm delivery, SGA, and still birth as compared to HIV-infected women who started ART during pregnancy and those who never received ART [14]. Further, among women initiating antiretroviral therapy in pregnancy use of triple-drug ART was associated with higher odds of preterm, SGA, and stillbirth as compared to ZDV monotherapy. The leading mechanistic hypotheses for this relationship are that triple-drug ART increases risk of hypertension and preeclampsia or there may be a cytokine-mediated effect directly from HAART [15-18]. As a result, the roll-out of Option B+ and triple-drug therapy for all HIV-infected pregnant women may increase the risk of adverse birth events for both current and future pregnancies.

**1.3 Vitamin D Sources and Levels among Adults and Infants Tanzania:** Vitamin D is a steroid hormone found in two major forms cholecalciferol (vitamin D3) and ergocalciferol (vitamin D2), which can be acquired through nutritional sources and from cutaneous synthesis by way of sun exposure [19]. Dietary sources are not regularly consumed in most parts of the world; hence exposure to sunlight is the primary source of vitamin D. Vitamin D3 is formed in the skin from exposure to ultraviolet B radiation at wavelengths of 290–315 nm [20,21]. After vitamin D3 is formed cutaneously, it enters the liver and undergoes hydroxylation to form serum 25- hydroxyvitamin D (25(OH)D), the circulating storage form of vitamin D. Circulating 25(OH)D then enters the kidney to undergo another hydroxylation reaction and form the metabolically active form of vitamin D, calcitriol (1,25(OH)2D) [22]. Contributors to low vitamin D levels in tropical locations include: staying indoors, dark skin pigmentation, religious coverings, and low intake of dietary sources [23]. Vitamin D levels for fetus depend on maternal vitamin D status during pregnancy as umbilical cord blood 25(OH)D concentrations are usually between 50% and 80% of the maternal levels [24]. After birth, the level of vitamin D for an exclusively breastfeeding infant will depend on sunlight exposure and vitamin D intake from breast milk [25,26]. The milk of healthy lactating women contains relatively small amounts of 25(OH)D and is usually considered insufficient to prevent vitamin D deficiency in exclusively breast-fed infants if sunlight exposure is limited [27, 28]. We have conducted three large vitamin D assessments in Tanzania and found 39% of HIV-infected pregnant women at 12-27 weeks gestation, 53% of HIV-infected men and non-pregnant women initiating ART, and 90% of HIV-exposed uninfected infants had vitamin D levels <32 ng/mL at 4-6 weeks of age.

**1.4 Vitamin D and HIV Progression:** Five published observational prospective cohorts of vitamin D and HIV progression have published to date and only one of these studies was conducted among pregnant women by our group in Tanzania [29]. The first small cohort study conducted in Norway among men and non-pregnant women before ART was available found that HIV-infected adults with low 1,25(OH)2D levels had significantly decreased survival time compared to individuals with normal levels [30]. The EUROSIDA cohort, comprised of HIV-infected adults (non-pregnant) in 31 European countries, Israel, and Argentina found individuals in the highest tertile (>20 ng/mL) of vitamin D had increased incidence of all-cause mortality and AIDS diagnoses as compared with individuals in the lowest tertile [31]. We also conducted a preliminary study among men and non-pregnant women initiating ART in Tanzania and found 25(OH)D levels <20 ng/mL were significantly associated with increased mortality as compared to those with >30 ng/mL (HR: 2.00; 95% CI: 1.19-3.37; p=0.009), and there was some indication of an attenuated association for those with vitamin D levels 20-30 ng/mL (HR: 1.24; 95% CI: 0.87-1.78; p=0.240) [32]. Further, 25(OH)D <20 ng/mL was significantly associated with incident pulmonary TB, incident oral thrush, wasting, and >10% weight loss [96].

**1.5 Maternal Vitamin D and Birth Outcomes:** There have been many observational studies of maternal vitamin D status and birth outcomes. A recent meta-analysis of 24 observational studies among HIV-uninfected women found that 25(OH)D levels <20 ng/mL were significantly associated with SGA (OR: 1.52; 95% CI:1.08-2.15) and preterm delivery (1.58; 95% CI: 1.08-2.31) [33]. In our preliminary study of HIV-infected pregnant women in Tanzania there was no significant association of vitamin D levels with birth outcomes, but SGA appeared to be elevated among those with 25(OH)D <30 ng/mL (RR: 1.25; 95% CI: 0.82–1.90) [29]. Despite the supportive evidence from observational studies, only a few small randomized trials have examined the efficacy of maternal vitamin D supplementation on birth outcomes [34]. Only two small vitamin D supplementation trials with data on SGA have been conducted. The first trial was conducted by Brooke in 1980 among 126 pregnant Asian women living in the UK and found that 1000 IU/day vitamin D2 appeared to decrease risk of SGA (RR: 0.54; 95% CI: 0.26-1.10) [35]. The second small RCT conducted by Yu among 180 pregnant women in the UK also found some indication that 200,000 IU vitamin D or a daily supplement of 800 IU vitamin D from 27 weeks until delivery decreased risk of SGA as compared to placebo (RR: 0.84; 95% CI: 0.41-1.71) [36]. As for preterm delivery (<37 weeks), there have been two trials of vitamin D supplementation that found no indication of an effect . Further, a recent trial of 35,000 IU/week of vitamin D3 versus placebo conducted in Bangladesh also found no difference in mean gestational age at delivery [37]. There have been four small RCTs conducted examining the effect of vitamin D supplementation on birth weight [37-40]. A pooled analysis of the 3 trials with data on low birth weight (<2500g) found combined daily or bolus vitamin D supplementation significantly decreased the risk of low birth weight (RR: 0.40; 95% CI: 0.23-0.71) [34]. A recent trial conducted in Iran randomized pregnant women 24-26 weeks gestation with 25(OH)D <30 ng/mL to 50,000 IU vitamin D3/week for 8 weeks or 400 IU vitamin D3/day and the mean birth weight was significantly higher in the 50,000 IU arm (mean 3429g vs. 3259g; p=0.01)[40]. No trials have been conducted among HIV-infected pregnant women, who may experience a greater effect on birth outcomes due to the immunomodulatory benefits of vitamin D3.

**1.6 Maternal Vitamin D and Child Linear Growth:** The beneficial effect of vitamin D on bone health and regulation of calcium metabolism are well documented; however, trials of vitamin D supplementation on linear growth are sparse and short in duration. There have been two small RCTs of maternal vitamin D supplementation in pregnancy and child growth. In the Brooke trial in the UK there was no significant difference in infant height up to 6 months of age for supplemented mothers, but by 1 year the infants of mothers who received 1,000 IU/day vitamin D2 grew 27.9 cm as compared to 24.6 cm in the placebo (p<0.001) [35]. In the Roth, et al. trial in Bangladesh, infants of mothers who received 35,000 IU vitamin D3/wk had significantly increased LAZ on average during the first years (mean difference LAZ: 0.41; p<0.01), and reduced risk of stunting (17% vs 31%) as compared to placebo [36]. Results from observational studies examining the association of vitamin D levels during pregnancy and child growth are mixed [33]. In our preliminary study of vitamin D levels during pregnancy among HIV-infected women, infants whose mother had a 25(OH)D level of <32 ng/mL at 12-27 weeks gestation had increased risk of stunting during the first two years of life (RR: 1.29; 95% CI: 1.05-1.59) [29]. Studies in The Gambia and India also found no association of maternal vitamin D levels with child linear growth, but a study in the UK found significantly increased height at 9 months of age with increase maternal vitamin D levels during pregnancy after taking in account birth length [41]. Maternal vitamin D supplementation during lactation also improves vitamin D status of the breastfeeding child. Hollis et al, found that infants of mothers receiving 4,000 IU/day vitamin D2 during lactation increased infant 25(OH)D from a mean of 13.4 ng/mL at 1 month of age to 30.8 ng/mL three months later [42]. Very few studies have investigated the association of maternal or infant 25(OH)D level during lactation on growth. We conducted a study of vitamin D among a cohort of 884 HIV-infected pregnant women (not receiving ART) and followed mothers and infants until 2 years post-partum [52]. We determined women with 25(OH)D <32 ng/mL had 1.25 times (95% CI:

1.05-1.50) the risk of reaching WHO HIV disease stage III or greater and the benefits appeared to increase up to 70 ng/mL [52]. We also found maternal 25(OH)D <32 ng/mL was also associated with maternal underweight, maternal anemia, and child stunting [52]. Our preliminary unpublished study examining 25(OH)D at 4-6 weeks for HIV-exposed uninfected Tanzanian infants on child growth found there was no association of infant 25(OH)D with infant LAZ any point during the first 2 years of life.

**1.7 Adverse Effects of Vitamin D Supplements:** In terms of safety, the only absolute contraindications to vitamin D supplementation are vitamin D toxicity or allergy; though there are no reports of acute allergic reactions to vitamin D in published literature [43]. Vitamin D intoxication cases due to supplementation have been sporadically reported over the last few decades with the most recent severe cases due to supplement manufacturer and labeling errors [44]. The toxic dose of vitamin D is estimated to be greater than 100,000 IU / day for at least 1 month [45]. Excessive vitamin D consumption can also cause hypercalcemia, serum calcium >2.6 mmol/L [46]. If hypercalcemia goes undetected calcification of internal organs, especially the kidneys can occur; however, to indict vitamin D as the cause of hypercalcemia 25(OH)D levels usually need to increase above 150 ng/mL, which requires most adults to consistently take in excess of 10,000 IU vitamin D per day for months or even years [47,48]. Acute symptoms of hypercalcemia can include: nausea, vomiting, excessive thirst, anorexia, symptoms of kidney stones, and confusion [49,50. The Institute of Medicine recently doubled the upper safety limit for vitamin D supplements for pregnant and lactating women to 4,000 IU daily [51]. Nevertheless, it should be noted that a 30 minute dose of sunshine is estimated to produce up to 10,000 IU of vitamin D [50].

**1.8 Preliminary Studies of Vitamin D in Tanzania**: We have conducted three large vitamin D assessments in Tanzanians among a) HIV-infected pregnant women who did not receive ART, ii) HIV-infected men and non-pregnant women initiating ART, and iii) HIV-exposed uninfected infants at 4-6 weeks of age. Each of these studies lends strong support for investigating the efficacy of vitamin D supplements for HIV-infected mothers receiving ART.

**a) Prospective cohort study of HIV-infected pregnant women:** In this cohort study we assessed 25(OH)D at 12-27 weeks gestation for 884 HIV-infected pregnant women (not receiving ART) and followed mothers and infants until 2 years post-partum. We determined women with 25(OH)D <32 ng/mL had 1.25 times (95% CI: 1.05-1.50) the risk of reaching WHO HIV disease stage III or greater and the benefits appeared to increase up to 70 ng/mL. We also found maternal 25(OH)D <32 ng/mL was also associated with maternal underweight, maternal anemia, and child stunting. There was also some indication low maternal 25(OH)D was associated with increased risk of SGA infants, but results were not statistically significant [52].

**b) Prospective cohort study of HIV-infected men and non-pregnant women**: In this study we assessed vitamin D at ART initiation for 1103 Tanzanian adult men and non-pregnant women. We found 25(OH)D levels <20 ng/mL were as associated with twice the risk of mortality as compared to levels >30 ng/mL (HR: 2.00; 95% CI: 1.19-3.37; p=0.009). In this study 25(OH)D <20 ng/mL at ART initiation was associated with incident pulmonary TB, incident oral thrush, wasting, and >10% weight loss. As a result, this study shows that even in the presence of triple-drug ART, vitamin D appears to have a strong impact on HIV disease progression.

**c) Prospective cohort study of HIV-exposed uninfected infants**: In an unpublished study, we assessed 25(OH)D at 4-6 weeks of age for HIV-exposed uninfected infants and prospectively followed infants until 24 months of age. In this study there was no association of infant 25(OH)D level at 4-6 weeks with child linear growth or stunting. Nevertheless, infant 25(OH)D levels <10 ng/mL were associated with increased incidence of wasting [weight-for-height <-2 z-score] (HR: 1.71; 95% CI: 1.20-2.43; p<0.01) and oral candidiasis. There was also some indication infants with 25(OH)D levels >30 ng/mL may experience increased mortality; however, there is high risk of residual and unmeasured confounding among this group consisting of <10% of infants in our sample.

**2.0 RATIONALE/PROBLEM STATEMENT**

HIV-infected pregnant women receiving antiretroviral therapy in Tanzania and sub-Saharan Africa experience high rates of mortality, morbidity, and adverse birth outcomes and their children also exhibit poor linear growth. Vitamin D has been shown to be a potent immunomodulator with effects on both adaptive and innate immune responses. As a result, HIV-infected pregnant women with higher levels of vitamin D may better control HIV replication or opportunistic infections, which may in turn improve birth and child health outcomes. We have also previously shown ~50% of HIV-infected pregnant women and ~90% of infants born to HIV-infected women have vitamin D deficiency/ insufficiency in Dar es Salaam, Tanzania.

To date no trials of maternal vitamin D supplementation have been conducted for HIV-infected pregnant women.

**3.0 OBJECTIVES**

The overall objective is to conduct a randomized, double-blind, placebo-controlled trial to determine the effect of maternal vitamin D3 oral supplements containing 3,000 IU starting during the second trimester and continued until 12 months post-partum on maternal and child health. Below are the primary and secondary aims of the trial.

**Primary Aims**

1) To determine the effect of daily maternal vitamin D3 supplementation (3,000 IU daily) on maternal HIV disease progression or death compared to placebo among HIV-infected pregnant women.

2) To examine the effect of daily maternal vitamin D3 supplementation on risk of infant small-for-gestational age (birth weight <10th percentile for gestational age).

3) To determine the effect of daily maternal vitamin D3 supplementation on the risk of child stunting (lengthfor- age z-score <-2) at 12 months of age.

**4.0 METHODOLOGY**

**4.1 Design** A randomized, double-blind, placebo-controlled trial to determine the effect of maternal vitamin D3 oral supplements containing 3,000 IU taken from the second trimester of pregnancy until 12 months post-partum. Infants will not receive any supplements.

**4.2** **Eligibility Criteria:**

*4.2.1 Inclusion criteria:* HIV-infected pregnant women and their children who are eligible for the trial will meet all the following inclusion criteria:

(a) Women aged ≥18 years old,

(b) HIV-positive,

(c) receiving antiretroviral therapy (initiating or already receiving),

(d) pregnant and of 12-27 weeks gestation (Second Trimester),

(d) calcium levels in the normal physiologic range (≤2.6 mmol/L),

(e) intending to stay in Dar-es-Salaam for 2 years after enrollment,

(f) not enrolled in any other clinical trial,

(g) provide informed consent.

*4.2.2 Exclusion criteria include:*

HIV-infected Pregnant women and their children who are NOT eligible for the trial will meet at least one of the following exclusion criteria:

(a) Girls (children) aged <18 years old,

(b) HIV-negative,

(c) Not receiving antiretroviral therapy,

(d) Outside the 12-27 weeks gestation window,

(d) calcium levels greater than the normal physiologic range (>2.6 mmol/L),

(e) intends to live outside Dar-es-Salaam during the 2 years after enrollment,

(f) enrolled in any other clinical trial,

(g) does NOT provide informed consent.

**4.3 Study sites:** Participants will be enrolled designated PMTCT clinics: Mnazi Mmoja (AKC), Buguruni, Mbagala Rangi Tatu, Magomeni, Tandale, Mbagala Round Table and Sinza PMTCT clinics. These clinics for the proposed trial have the highest client numbers and receive passive referrals from other satellite health centers. We have operated at these same locations for other clinical trials and we maintain adequate space and have excellent working relationships with physicians and nurses.

**4.4 Screening and Enrollment Procedures:** Prior to randomization potential participants (HIV-infected pregnant women) will attend a screening visit during which the study team will document eligibility, and seek informed consent for calcium screening. (a) The first screening visit will be incorporated with standard HIV and pregnancy testing protocols within the MDH PEPFAR program. HIV status will be confirmed using two licensed rapid assays, even if the participant was previously determined to be HIV-positive at a non-study testing site. Discordant results will be confirmed using an ELISA. All women will also receive a urine pregnancy test to confirm pregnancy. Gestational age will be assessed through report of last menstrual period. (b) Second visit: A second visit will occur 1 week later (maximum of 21 days after) and the period between the first and second visit will allow mothers to consider the matter of participation in the study further and discuss the study with family members, thereby reducing the number of defaulters once randomization has taken place. At this visit, we will also review the serum calcium test drawn at visit #1; women with hypercalcemia (albumin-adjusted calcium >2.6 mmol/L) will be excluded from randomization procedures and provided appropriate clinical management. Once a normal screening calcium level is confirmed, the subject’s eligibility criteria will be confirmed and written consent for participation in the trial will be sought. Individuals enrolled in the trial will not be eligible for other clinical trials.

**4.5 Experimental Regimens and Randomization Procedures:** Pregnant women will be randomized to receive of one of two regimens: (a) vitamin D3 (cholecalciferol) oral supplements containing 3,000 IU taken daily from randomization (second trimester) until 12 months post-partum or (b) placebo taken daily from randomization until 12 months post-partum. There will be no discernible difference between the vitamin D and placebo tablets in appearance or taste. A list from 1 to 2300 will be prepared according to a randomization sequence in blocks of 10 and stratified by follow-up clinic. All study staff will be blinded to the patient’s randomization group. At randomization, each eligible person will be assigned to the next numbered regimen at each clinic site, which will correspond to a set of supplement bottles. Experience has shown that unblinding, whether real or based on an impression or perception that develops among subjects or staff, is more likely to occur if regimens are color-coded or bear simple numeric codes. To minimize this risk, we will provide the regimen in bottles labeled with the participant’s identification numbers, and will make active tablets and placebo indistinguishable, so that neither the subjects nor the investigators can identify which group of subjects is randomized to the same regimen.

**4.6 Regimen Selection**: The regimen was selected in order to increase and sustain 25(OH)D levels to >32 ng/mL for nearly all pregnant women in the active vitamin D arm, while having very minimal risk of hypercalcemia events. The landmark safety and effectiveness trial of vitamin D3 supplementation during pregnancy by Hollis and colleagues randomized US women with a singleton pregnancy at 12–16 weeks’ gestation to receive 400, 2000, or 4000 IU vitamin D3/day until delivery [37]. This study found that at delivery the proportion of women reaching 32 ng/mL was comparable for women in the 4,000 IU arm (84%) and the 2,000 IU (80%) but was significant reduced for the 400 IU (52%) arm. There were no differences on any safety measure and the DSMB determined no hypercalcemia events were attributed to vitamin D supplementation or circulating 25(OH)D levels. Based on these and other older data the Institute of Medicine increased the tolerable upper intake limit (UL) of vitamin D3 to 4000 IU/day during pregnancy and lactation [51]. We decided to utilize 3,000 IU per day since the studies the IOM used to set the 4,000 IU tolerable upper limit were not conducted among pregnant women residing equatorially, some of whom may often be in the sun. We restricted enrollment for the proposed trial to 12-27 weeks gestation due to a complete lack of safety data on the use of 3,000 IU daily supplements units before 12 weeks of age, which is the period of early organogenesis. Vitamin D3 supplements (cholecalciferol) were chosen over vitamin D2 (ergocalciferol) supplements, since vitamin D3 appears to be more effective in increasing and sustaining high levels of circulating 25(OH)D.

**4.7 Prenatal Follow-up:** Each enrolled pregnant woman (12-27 weeks) will be seen in the research clinic once a month until the 32nd week of pregnancy, every woman will have a 32nd week visit, and then have a visit once every 2 weeks until the 36th week, and then once every week until delivery. Pregnant mothers will receive a general physical exam and morbidities occurring since the last visit will be documented. This follow-up visit schedule has been effective in identifying maternal morbidity and has been acceptable to mothers if transportation costs are reimbursed for study visits that are not standard PMTCT visits.

**4.8 Delivery Assessment:** Women will be asked to deliver at the same hospital where they are being followed up at, where study staff will also be located. Asking mothers to give birth at specific study clinics has proved to be effective in capturing births in past trials and very acceptable to mothers as the relationships and quality care given by study personnel is highly valued. Study nurses/midwives will be available round-the-clock to attend to study women, document details of delivery, and schedule post-natal appointments. At delivery, women will be asked about any pregnancy complications since the last study visit and the duration of each stage of labor and complications of labor will be recorded. Immediately after delivery trained research midwives will determine Apgar scores at one and five minutes. Length, weight, head circumference, chest circumference, and mid-upper arm circumference (MUAC) of the infant will also be taken. We will keep a record of the Expected Date of Delivery, and if the participant does not come to the study clinic or present in labor up to 3 days after her due date, she will be visited at home; if she was found to have given birth, the child will be examined by the visiting nurse, and the mother will be encouraged to come to the study clinic for a complete examination by a study pediatrician and collection of anthropometrics. If the mother and child are not able to come to clinic, the study nurse will document morbidity, take anthropometric measurements using mobile equipment and record birth complications in the home.

**4.9 Postnatal Follow-up:** In order to integrate into standard PMTCT visits, the mother/child pair will be followed 6 weeks post-partum, at 2 months and every month thereafter until 12 months post-partum. At each of these visits the mother and child will receive a physical exam, morbidities occurring since the last visit will be documented by a nurse, and both mother and child will undergo anthropometric measurements. We specifically chose monthly follow-up during the first 12 months post-partum in order to encourage exclusive breastfeeding for the first 6 months and accurately ascertain breastfeeding method (exclusive, predominant, partial, none). Breastfeeding is the primary source of vitamin D for the child and as a result accurate data on the duration and type of breastfeeding will be important to examine as an effect modifier in the analysis of child growth. The mother and child will receive a complete physical exam, document morbidities since the last visit, and have anthropometrics measured. All mothers and children will be discharged from the study at 12 months of age.

**4.10 Standard of Care:** All study participants will be provided with HIV care and treatment that adhere to Tanzanian national guidelines. The first-line ART regimen for HIV-infected pregnant women is tenofovir

(TDF) + lamivudine (3TC) + efavirenz (EFV) and alternative first-line regimens include AZT+3TC+EFV or

AZT+3TC+NVP if not well tolerated. Mothers return to the clinic monthly to meet with a nurse counselor and pick up medications, and see a physician. Laboratory assessments occur at 4-monthly intervals or under the discretion of a clinician and include: CD4 T-cell count and hematology testing. Although viral load assays are routinely performed in the developed countries, due to high cost, the MDH program only perform viral loads on individuals with suspected treatment failures. Prophylaxis for opportunistic infections is provided in line with the national HIV care guidelines. Malaria prophylaxis is provided with two dose of sulphadoxine pyremethamine at 20-24 weeks of gestation and one at 28-32 weeks. Daily iron (60 mg elemental) and folate (400 mcg) supplementation is established as standard prenatal care practice in many developing countries including Tanzania and will be provided. All HIV-positive women will receive single RDA multivitamins including vitamins B-complex, C and E as per standard of care. Adult patients will receive co-trimoxazole.

**5.0 SAMPLE SIZE CALCULATIONS**

Power calculations are based on enrollment of 2300 HIV-infected pregnant women with 1:1 randomization to the vitamin D3 or placebo regimen. Power for the primary aims were calculated with a nominal Type I error rate (alpha) of 0.05. We also assumed a 90% retention rate of HIV-infected mothers until study discharge at 1 year post-partum, 90% retention of birth outcomes, and 85% retention of infants 12 months post-partum to take in account for fetal loss and loss to follow-up. Mothers who experience fetal loss will remain in the trial to assess HIV progression outcomes until 12 months after the estimated due date.

| Primary #1: HIV progression and death (n=2300 w/ 90% retention) | | | | Primary #2: SGA (n=2300 w/ 90% retention) | | | | Primary #3: Child Stunting (n=2300 w/ 85% retention) | | | |
| --- | --- | --- | --- | --- | --- | --- | --- | --- | --- | --- | --- |
| Cum. Incidence Placebo | Relative Risk Vitamin D versus Placebo | | | Cum. Incidence Placebo | Relative Risk Vitamin D versus Placebo | | | Cum. Incidence Placebo | Relative Risk Vitamin D versus Placebo | | |
|  | RR= 0.70 | RR= 0.80 | RR= 0.85 |  | RR= 0.65 | RR= 0.70 | RR= 0.75 |  | RR= 0.60 | RR= 0.70 | RR= 0.80 |
| 35% | >99.9% | 99.80% | 66.60% | 10% | 98.4% | 93.4% | 81.2% | 15% | >99.9% | 98.8% | 78.4% |
| 50% | >99.9% | >99.9% | 89.7% | 12% | 99.5% | 96.9% | 88.3% | 20% | >99.9% | >99.9% | 90.3% |
| 65% | >99% | >99% | 99.1% | 14% | 99.9% | 98.7% | 93.0% | 25% | >99.9% | >99.9% | 96.3% |

Power for the primary outcomes is based on a 2-sided test of proportions and a z-statistic using the asymptotic variances of the observed proportions. The table above displays the power of the study to detect the relative risk (RR) of the outcome in those randomized to vitamin D compared to placebo varying the cumulative incidence of the endpoint in the placebo arm. The proposed trial with 2300 HIV-infected pregnant women will have good power to detect a RR of 0.85 on the composite HIV progression and death outcome, even if the cumulative incidence is as low as 50% in the placebo group. In a previous multivitamin trial (not including D) among HIV-infected men and women on HAART in the same setting, the risk of HIV progression or death was 72% over 2 years; however, HIV-infected pregnant women tend to be healthier as compared to the general ART population (particularly using Option B+), but we do not expect the risk of progression to go lower than 65%.

All previous maternal vitamin D supplementation trials with data on birth and child growth outcomes have <200 participants and as a result have very imprecise effect estimates. A pooled estimate of existing trials in our recent meta-analysis indicated vitamin D3 supplementation has a potentially strong effect on SGA with a relative risk of 0.67 (95% CI: 0.40-1.11), but results were not statistically significant. We will have good power to detect a relative risk on SGA as low as 0.75 if the risk of SGA in the placebo is below expectations at 10%. As for child growth, in a recent RCT (not including vitamin D) we conducted among children of HIV-infected women in Dar-es-Salaam, the risk of stunting at 12 months was 20%. As for the potential effect size for child stunting, a recent small randomized trial of maternal vitamin D3 supplementation during the 3^rd^ trimester in Bangladesh found a large effect size on child stunting (Odds ratio for stunting during first year 0.45, 95% CI:0.20-0.996). In the proposed trial we will have adequate statistical power even if both the incidence of stunting and the effect size are unexpectedly smaller at 15% and RR=0.80. It is important to note that none of the trials used in the above power calculations included HIV-infected women who may exhibit greater benefits from vitamin D supplementation as compared to HIV-uninfected women due to high morbidity rates and our preliminary observational data suggest additional benefits in obtaining 25(OH)D levels above 32 ng/mL for HIV-infected pregnant women.

**6.0 DATA MANAGEMENT AND ANALYSIS**

**Data Management:** We have developed a stringent data management system for use in our ongoing trials. All questionnaires and laboratory forms that accompany specimens for analysis are tracked using a 'batch' system. All forms of a particular type filled over a certain period of time are processed together as a batch. The processing steps include several stages of review and editing for checking completeness and consistency, double data entry, supervisory checks of the inconsistencies discovered at second data entry. Data will be sent to Harvard Channing Laboratory every 6 months where the files will be converted to SAS datasets and analyzed for DSMB meetings. Quality assurance and randomization checking will be undertaken in Boston and results sent to Dar es Salaam.

Data Analysis: An intent-to-treat analysis will be used as the primary analytic strategy for all analyses. We will also investigate effect modification by third variables and baseline imbalances in risk factors (concomitant) by randomization group for mortality, SGA, and stunting.

**Primary Aim #1- HIV disease progression or death:** We will test differences in the proportion of participants who experience a HIV disease progression event or die among HIV-infected pregnant women randomized to the vitamin D supplementation group as compared to the placebo.

**Primary Aim #2- SGA:** We will similarly test differences in the proportion of infants who are small-for-gestational age between treatment arms.

**Primary Aim #3- Child Stunting at 12 months:** We also will test differences in the proportion of infants who are stunted at 12 months of age between randomization groups

**7.0 PERSONNEL**

Below please find a brief description of the study personnel. We have also included CVs at the end of the document.

**Professor Karim Manji, MD, Principal Investigator** will devote 15% effort and will manage the subcontract in Tanzania, provide technical and scientific input for all aspects of the trial and will be in charge of the fiscal management of the subcontract. He will also provide continuing training and supervision to the nurses and physicians and also consult on clinical management of trial participants. Similar arrangements in previous trials were successful in achieving efficient progress in the field and prompt resolution of any problems encountered.

**Professor Said Aboud, MD**, **PHD, Investigator** will devote 10% effort and will serve as the lab supervisor. He will provide uniformity and quality assurance to the overall laboratory aspects. This includes monitoring the quality of work of lab technicians when specimens are being handled, supervise the storage of specimens, manage the inventory and ordering of lab supplies, and oversee the maintenance schedules of all lab equipment. Dr. Aboud has been the lab manager in previous multivitamin trials conducted in Tanzania by our group. He will also contribute to data analysis and preparation of manuscripts.

**Dr. Fadhlun Albeity, M.D.,Investigator** will devote 10% effort and will serve as the clinical supervisor. He will work closely with the medical staff and advise on the quality of the medical care, prescription of appropriate drugs and training related to the study. He will meet regularly with the physicians and research staff to assure high quality clinical services. He will also provide support of field operations and data management, and will also contribute to data analysis and manuscript preparation.

**Harvard**

**Dr. Wafaie Fawzi, M.B.B.S., M.P.H., M.S., DR.P.H., Principal Investigator,** will oversee the scientific, administrative aspects of the study including the maintenance of data collection and management systems, follow-up procedures, and the conduct of statistical analysis. He will also oversee the budget administration of the project. He spends about 2 months of every year in Tanzania, where he will meet regularly with the colleagues at MDH to jointly monitor the progress of the work.

**Dr. Christopher Sudfeld, ScD, Principal Investigator** oversee the scientific, field operations, enrollment and follow-up progress, data quality, and statistical analysis of the trial. Dr. Sudfeld has significant experience with coordination of field aspects of research studies in Tanzania. Dr. Sudfeld will perform weekly analyses to monitor enrollment and follow-up progress and give data-driven feedback to field teams. He will also conduct regular quality control analyses of the data and will give provide information to staff in Dar es Salaam who have statistical questions in need of a resolution.

**Dr. Christopher Duggan, M.D., M.P.H.,** Investigator: will oversee the clinical and safety aspects of the trial. This will include assisting with training and overseeing quality of maternal HIV and child clinical care during pregnancy, delivery, and post-partum periods. Dr. Duggan spends 1 month per year in Tanzania and will supervise training of clinical research staff and will provide re-trainings to maintain standard of

care.

**Dr. Molin Wang, PhD, Biostatistician**, will oversee data management and all statistical analyses of the trial. She has extensive experience in design and analysis of clinical trials and expertise in survival analysis and measurement error correction. Dr. Wang will spend 2 weeks per year in Dar es Salaam to monitor ongoing data management procedures. She will also conduct regular quality control analyses of the data in

Boston and will give provide information to staff members in Dar es Salaam who have statistical

questions in need of a resolution.

**8.0 ETHICAL CONSIDERATION**

Ethical considerations for any clinical trial are always of paramount importance. We are proposing a randomized, double-blind, placebo controlled trial of vitamin D3 to evaluate the safety and efficacy of maternal vitamin D3 supplementation among HIV-infected women and their children. The study will be under review by the Institutional Review Board at Harvard School of Public Health and by the National Institute of Medical Research (NIMR) in Tanzania

**8.1 Study Population:** We will recruit 2300 HIV-infected pregnant women receiving PMTCT care within the MDH PEPFAR program. Women with above the normal range of calcium at screening will not be randomized in the trial nor will those who do not consent for enrollment. These women will be followed at prenatal study clinic visits, at delivery, and with their child at study visits until 12 months post-partum.

**8.2 Recruitment and Consent Ethics:** Written informed consent will be sought from all HIV-infected pregnant women. In the process of seeking informed consent, the research nurse will make it clear to every patient that participation in the research is voluntary and that she could stop participating at any point after consent is granted without any penalty. In addition to the study investigators, a person at MDH who is not part of the trial will be designated as someone to whom the patients may go with any concerns regarding the study, or if they would like to terminate their participation in the trial.

**8.3 Procedures for Minimizing Risks:** Each enrolled pregnant woman (12-27 weeks) will be seen in the research clinic once a month until the 32nd week of pregnancy, every woman will have a 32nd week visit, and then have a visit once every 2 weeks until the 36th week, and then once every week until delivery. Mothers will have a study visit during labor and delivery. After delivery the mother/child pair will be followed 6 weeks post-partum, at 2 months and every month thereafter until 12 months post-partum. Standard of care according to national guidelines will be provided for the mother and child, including diagnosis and treatment of opportunistic infection and other conditions. Psychosocial and nutrition counseling for the mother will be provided. A risk lies in the social stigma that would be associated with loss of confidentiality. Confidentiality will be strictly observed. It will be up to the individual patient to decide whether or not to tell a spouse or other relative the results of HIV status; this information will never be revealed to or discussed with third parties. To further protect confidentiality, study subjects will attend a study clinic that is located within the larger routine clinics; we will use the same clinic flow pattern, same clinic cards, and health education sessions. Our research staff will dress in the same way as the clinic nurses. A study nurse and a study physician will be available at the study clinic to see clients who prefer to have their routine research visits at ordinary clinic hours and even on Saturdays. Examination and counseling of clients will also be carried out in private cubicles. Laboratory specimens will be taken to the laboratory by one of our research staff and are given only to authorized personnel working in the laboratory.

**8.4 Data and Safety Monitoring Plan:** A Data and Safety Monitoring Board (DSMB) has also been formed to direct data analyses for assessing treatment effects during the trial. The trial would be stopped if there is early indication of significant differences between the treatment groups. Members who have accepted to serve on the DSMB include: Dr. Michael Holick (Professor of Medicine, Physiology and Biophysics at Boston University School of Medicine); Dr. Salim Abdulla(Chief Executive Director at Ifakara Health Institute); Dr. David Shapiro (Biostatistician at Harvard School of Public Health); and Professor Gibson Kibiki (Associate Professor at Kilimanjaro Christian Medical University College).

**8.5 Potential Benefits:** A direct benefit to the study participants is that all participants will have access to the study clinics throughout the study period and will be encouraged to use the clinics at any time if they or their children require medical attention. Further, vitamin D supplementation may be prolong or improve the quality of life for the mother and/or child. The knowledge gained from this study may identify a low cost intervention to better treatment for HIV-infected pregnant mothers in resource-limited settings.

**8.6 Importance of knowledge to be gained:** Vitamin D3 supplementation may be an attractive intervention to improve PMTCT care in resource-limited settings given low levels of vitamin are common in HIV-infected pregnant women, ART can reduce vitamin D levels by altering vitamin D metabolism, and vitamin D supplements are known to be effective in improving vitamin D status. Evidence from this randomized trial is urgently needed since ART coverage is rapidly expanding globally and treatment programs are in need of interventions to prolong and improve the quality of life for HIV-infected pregnant women and their children.

**9.0 DSMB MEMBERS**

It is anticipated that the first review will occur approximately 6 months after the first subject enrolls, with subsequent reviews 6 monthly thereafter. Members of the DSMB provide varied and complementary expertise and include: Members who have accepted to serve on the DSMB include: Dr. Michael Holick (Professor of Medicine, Physiology and Biophysics at Boston University School of Medicine); Dr. Salim Abdulla (Chief Executive Director at Ifakara Health Institute); Dr. David Shapiro (Biostatistician at Harvard School of Public Health); and Professor Gibson Kibiki (Associate Professor at Kilimanjaro Christian Medical University College). It is anticipated that the first review will occur approximately 6 months after the first subject enrolls, with subsequent reviews yearly thereafter. Each meeting will include an administrative review to assess accrual, retention, and the progress of the study. In addition, the DSMB will monitor the occurrence of any adverse effects; these will include clinical signs and results of laboratory investigations done within the context of the trial. The DSMB will define stopping rules at its first meeting. Additional reviews or an altered schedule may be instituted at the discretion of the DSMB. After each DSMB meeting, the committee will prepare and submit a report to the Principal Investigator, which will then be forwarded to the Institutional Review Boards at the Harvard School of Public Health and the Tanzanian National Institute or Medical Research (NIMR).

**10.0 LIMITATIONS OF THE STUDY**

**Limitations and Strengths of the Proposed Trial**

a)Retention: A major limitation in large trials is the operational difficulty in following up subjects for long periods of time. We have significant experience with trials in Tanzania in which we instituted a package of non-coercive incentives for pregnant women to continue follow-up and we expect a follow-up rate of 90%.

b) Adherence: We recognize that non-adherence decreases the statistical power of a study to detect any true effect of the regimens. In the proposed study we will monitor ingestion of pills by questioning the subjects, by pill count, and by measurement of plasma 25(OH)D levels in a sub-sample of subjects. We will attempt to maximize adherence of the study subjects by providing counseling and allowing the patients adequate time to think through the issues during the recruitment phase, thus selecting a study population that is interested and reliable. We will also counsel all women monthly to follow WHO breastfeeding guidelines for HIV-infected mothers, since breast milk is the primary source of vitamin D for the infant.

**11.0 DISSEMINATION OF RESULTS**

We plan to disseminate the results of the trial at appropriate local, Tanzanian national, and international levels. If it is found that vitamin D3 is effective in improving maternal or child health outcomes we will work with the Taznanian Ministry of Health to introduce maternal vitamin D3 as standard of care for HIV-infected pregnant women.
