# Supplementary material for: Vitamin D3 supplementation during pregnancy and lactation for women living with HIV in Tanzania: A randomized controlled trial
Source: PLoS Med. 2022 Apr 15;19(4):e1003973. doi: 10.1371/journal.pmed.1003973 (PMC9012360; doi:10.1371/journal.pmed.1003973)
Supplement: S1 Analysis Plan — (DOCX) [file pmed.1003973.s003.docx]

**Trial of Vitamins-5 (ToV5)**

Statistical Analysis Plan

Version 3.0

ClinicalTrials.gov identifier [NCT02305927](https://clinicaltrials.gov/ct2/show/NCT02305927)

1 Version history 3

2 Introduction 4

3 Specific aims 5

4 General approach to data analysis 5

5 Primary outcome definitions 6

6 Secondary outcome definitions 6

7 SAEs and safety outcomes 7

8 Statistical analysis 7

# Version history

| **Version number** | **Version date** | **Description of the completed editions** |
| --- | --- | --- |
| 1.0 | September 20, 2024 | First draft – IRB Protocol |
| 2.0 | Feb 3, 2017 | Clarified secondary outcomes and analysis included in Trials published protocol paper |
| 3.0 | October 28, 2019 | Finalized SAP before undertaking analysis –  i) Added sensitivity outcomes – Intergrowth SGA  ii) Clarified definition of stillbirth vs miscarriage by 28 week cut off per WHO  iii) Dropped analysis of follow-up CD4 due to lack of testing  iii) Clarified analysis to account for multiple gestation and addition sensitivity analysis for singletons |

# Introduction

Vitamin D has been shown to be a potent immunomodulator with effects on both adaptive and innate immune responses. Vitamin D appears to play a particularly strong role in control of intracellular pathogens by enhancing cell-mediated immunity, production of antimicrobial peptides, and phagocytic activity of macrophages. As a result, HIV-infected pregnant women with higher levels of vitamin D may better control HIV replication or opportunistic infections, which may in turn improve birth and child health outcomes. Our preliminary data, via an observational prospective cohort study conducted among HIV-infected pregnant women in Tanzania, found low vitamin D serum concentrations at 12-27 weeks gestation were associated with maternal HIV disease progression, maternal death, maternal anemia, and child stunting. These relationships persisted with statistical control for a comprehensive set of socio-demographic factors and markers of HIV disease severity; however, the risk of residual and unmeasured confounding is quite plausible.

The Trial of Vitamins-5 (ToV5) is an individually randomized, parallel group, double-blind, placebo-controlled trial of vitamin D3 (cholecalciferol) supplementation conducted among 2300 HIV-infected pregnant women receiving ART in Dar es Salaam, Tanzania (ClinicalTrials.gov identifier NCT02305927). The trial protocol was developed by collaborators at the Harvard T.H. Chan School of Public Health in the United States and Management and Development for Health (MDH) and Muhimbili University of Health and Allied Sciences (MUHAS) in the United Republic of Tanzania.

# Specific aims

## Primary Aims:

1) To determine the effect of daily maternal vitamin D3 supplementation (3,000 IU daily) on maternal HIV disease progression or death compared to placebo among HIV-infected pregnant women.

2) To examine the effect of daily maternal vitamin D3 supplementation on risk of infant small-for-gestational age (birth weight <10th percentile for gestational age).

3) To determine the effect of daily maternal vitamin D3 supplementation on the risk of child stunting at 12 months of age.

# General approach to data analysis

## Intention to treat (ITT) analysis

An intent-to-treat analysis will be used as the primary analytic strategy for all analyses.

Stratified randomization

The trial utilized a stratified randomization scheme by study clinic. As a result, all primary and secondary analyses will account for the randomization schema and adjust for country of enrolment by including fixed effects for country all analyses.

## Sensitivity analyses examining potential baseline imbalances

## We will also conduct a sensitivity analysis that adjusted for baseline factors that showed some degree of imbalance between treatment groups based on a p<0·20.

Sensitivity analysis SGA by Intergrowth-21st

A sensitivity analysis will be conducted for defining SGA (<10^th^ percentile) by Intergrowth standard

Sensitivity analysis limited to singleton births

A sensitivity analysis will be conducted among singleton births for all birth outcome and postnatal infant outcomes.

Effect modification of treatment effects

We will examine effect modification of any treatment effect by predefined baseline variables: maternal age, maternal body mass index, socioeconomic status, gestational age at randomization, CD4 T-cell count, hemoglobin concentration, WHO HIV disease stage, ART regimen, duration of ART, duration of exclusive and any breastfeeding, and trial regimen adherence. To assess the statistical significance of each interaction, we will use the likelihood ratio test for risk-ratio homogeneity for primary and secondary nonrepeatable binomial outcomes and the score test in the linear mixed-effects and generalized linear mixed models for repeatable binomial outcomes and continuous longitudinal secondary outcomes.

# Definitions of the Primary Outcomes

1) Maternal HIV Progression or death - Maternal HIV progression will be defined as any increase in WHO HIV disease stage or death from the WHO HIV stage at randomization.

2) Small for gestational age (SGA) will be defined as a birth weight less than the tenth percentile for gestational age by sex utilizing the Oken standard.

3) Child Stunting at 12 months will be defined as LAZ which is 2 or more standard deviations below the WHO child growth standard reference median.

# Definitions of Secondary Outcomes

1) post-randomization HIV viral load

2) ~~postrandomization maternal CD4 T-cell count~~ -DROPPED due to program stopping testing

3) postrandomization maternal immunologic biomarker levels (IL-2, IL-12, IFN-γ, and cathelicidin)

4) postrandomization maternal weight during pregnancy;

5) maternal depression and anxiety as assessed by the Hopkins symptoms checklist;

5) maternal hypercalcemia (serum albumin adjusted calcium > 2.6 mmol/L);

6)fetal death;

7) miscarriage;

8) stillbirth;

9) preterm birth;

10) birthweight;

11) low birthweight (<2500 g);

12) mother-to-child transmission of HIV;

13) infant mortality;

14) infant growth trajectory in the first year of life (LAZ, WAZ, and WLZ);

15) infant wasting (WLZ < –2);

16) infant underweight (WAZ < –2);

17) infant morbidities during the first year of life;

18) postrandomization infant cognitive, motor, and socioemotional development scores on the CREDI;

19) serum 25(OH)D levels in the mother and in the infant;

20) postrandomization maternal and infant PTH concentration.

# Serious Adverse Events and Safety Outcomes

The following SAEs will be monitored: death, fetal loss, unexpected life-threatening or suspected adverse event related to program procedure or participation. The predefined safety outcome is maternal hypercalcemia (serum albumin adjusted calcium > 2.6 mmol/L).

# Statistical analyses

**Primary outcomes**

1) Maternal HIV Progression - Log-rank test stratified by study clinic to assess differences in the incidence of maternal HIV progression or death by randomized arm. Hazard ratios also assessed with Cox models

2) Small for gestational age (SGA – 10^th^ percentile Oken) – Relative risks calculated with Generalized estimating equations with a compound symmetry working correlation matrix to used account for correlations due to multiple gestation and a log link and binomial variance function

3) Child Stunting at 12 months- Relative risks calculated with Generalized estimating equations with a compound symmetry working correlation matrix to used account for correlations due to multiple gestation and a log link and binomial variance function

**Secondary outcomes**

i) Time to event: Log-rank test stratified by study clinic to assess differences in the incidence of maternal HIV progression or death by randomized arm

ii) Generalized linear mixed models with random intercepts, compound symmetric covariance structures, and robust standard errors will be used for repeated binomial secondary outcomes. Infant analyses will take in account correlation for multiple gestation and a sensitivity analysis for singletons will be conducted.

iii) Continuous: linear mixed-effects models with a random intercept, a compound symmetric covariance structure, and robust standard errors. Infant analyses will take in account correlation multiple gestation.

iv) Maternal Hypercalcemia - Safety Fisher’s exact test will be used to evaluate differences in proportion of participants with incident maternal hypercalcemia between treatment groups
