# Supplementary material for: Vitamin D3 supplementation during pregnancy and lactation for women living with HIV in Tanzania: A randomized controlled trial
Source: PLoS Med. 2022 Apr 15;19(4):e1003973. doi: 10.1371/journal.pmed.1003973 (PMC9012360; doi:10.1371/journal.pmed.1003973)
Supplement: S1 Appendix — (DOCX) [file pmed.1003973.s004.docx]

**Table A.** Exploratory analysis of potential modifiers of the effect of randomized regimen on HIV progression or death from any cause

|  | Vitamin D_3_  n / N (%) | Placebo  n / N (%) | Hazard Ratio  (95% CI) | P-value for effect modification |
| --- | --- | --- | --- | --- |
| Overall – all participants | 166 / 1148 (14.5%) | 141/ 1151 (12.3%) | 1.21 (0.97-1.52) |  |
| *Subgroups* |  |  |  |  |
| Maternal age |  |  |  |  |
| 18 – 24 years | 22 / 180 (12.2%) | 20 / 189 (10.6%) | 1.25 (0.68, 2.28) | 0.44 |
| 25 – 34 years | 93 / 673 (13.8%) | 87 / 665 (13.1%) | 1.08 (0.81, 1.45) |  |
| 35+ years | 51 / 295 (17.3%) | 34 / 297 (11.5%) | 1.52 (0.99, 2.34) |  |
| Weeks gestation at randomization |  |  |  |  |
| 12-19.9 weeks | 82 / 509 (16.1%) | 62 / 492 (12.6%) | 1.29 (0.93, 1.80) | 0.60 |
| 20-27 weeks | 84 / 639 (13.2%) | 79 / 659 (12.0%) | 1.14 (0.84, 1.55) |  |
| Body mass index at randomization |  |  |  |  |
| <25.0 kg/m^2^ | 92 / 546 (16.9%) | 64 / 559 (11.5%) | 1.54 (1.11, 2.12) | 0.03 |
| ≥ 25.0 kg/m^2^ | 72 / 593 (12.1%) | 77 / 583 (13.2%) | 0.93 (0.67, 1.27) |  |
| Missing* | 2 / 9 (22.2%) | 0 / 9 (0%) | Not estimable |  |
| Household wealth |  |  |  |  |
| < Median | 81 / 588 (13.8%) | 67 / 560 (12.0%) | 1.15 (0.83, 1.59) | 0.67 |
| ≥ Median | 85 / 559 (15.2%) | 74 / 590 (12.5%) | 1.27 (0.93, 1.73) |  |
| Missing | 0 / 1 (0%) | 0 / 1 (0%) | Not estimable |  |
| WHO HIV disease stage at randomization |  |  |  |  |
| I | 155 / 1004 (15.4%) | 130 / 969 (13.4%) | 1.16 (0.92, 1.46) | 0.75 |
| II, III, IV | 11 / 144 (7.6%) | 11 / 182 (6.0%) | 1.33 (0.58, 3.05) |  |
| CD4 T-cell count at randomization |  |  |  |  |
| <350 cells per μL | 27 / 239 (11.3%) | 27 / 235 (11.5%) | 0.92 (0.54, 1.57) | 0.49 |
| ≥350 cells per μL | 39 / 305 (12.8%) | 33 / 302 (10.9%) | 1.22 (0.77, 1.94) |  |
| Missing | 100 / 604 (16.6%) | 81 / 614 (13.2%) | 1.33 (0.99, 1.78) |  |
| Timing of ART initiation |  |  |  |  |
| Before this pregnancy | 79 / 459 (17.2%) | 64 / 476 (13.5%) | 1.38 (0.98, 1.92) | 0.35 |
| During this pregnancy | 87 / 689 (12.6%) | 77 / 675 (11.4%) | 1.11 (0.82, 1.50) |  |
| Regimen adherence |  |  |  |  |
| <90% adherence | 69 / 614 (11.2%) | 74 / 680 (10.9%) | 1.06 (0.77, 1.48) | 0.37 |
| ≥90% adherence | 97 / 534 (18.2%) | 67 / 471 (14.2%) | 1.31 (0.96, 1.79) |  |

ART: Antiretroviral therapy, CI: confidence interval, HIV: Human immunodeficiency virus, WHO: World Health Organization

**Table B.** Cause of death for mothers stratified by treatment group

|  | Vitamin D_3_  N=6 | Placebo  N= 17 |
| --- | --- | --- |
| *Pregnancy-related death (pregnant or within 42 days postpartum)* |  |  |
| Severe anemia | 0 | 1 |
| Postpartum hemorrhage | 0 | 1 |
| Epilepsy | 0 | 1 |
| Cardiovascular disease /stroke | 1 | 0 |
|  |  |  |
| *Late pregnancy-related death (43 days -365 postpartum)* |  |  |
| Pulmonary tuberculosis | 1 | 1 |
| Diarrhea | 0 | 2 |
| Sepsis | 0 | 1 |
| Pneumonia - unspecified | 0 | 1 |
| Pneumocystis pneumonia | 0 | 1 |
| Malaria | 0 | 1 |
| Unspecified infection | 1 | 0 |
| Cardiovascular disease /stroke | 1 | 1 |
| Cancer | 0 | 1 |
| Liver failure | 0 | 1 |
| Motor vehicle accident | 1 | 0 |
| Unknown | 1 | 4 |

**Table C.** Effect of maternal vitamin D_3_ supplementation on small-for-gestational age livebirth and secondary birth and infant outcomes restricted to singletons

|  | Vitamin D_3_  n / N (%)  No. events / No. at risk (%)  or Mean (SD) | Placebo  No. events / No. at risk (%)  or Mean (SD) | Relative risk  (95% CI)  or Mean difference | p-value |
| --- | --- | --- | --- | --- |
| Fetal death | 66 / 1110 (6.0%) | 68 / 1107 (6.1%) | 0.97 (0.70, 1.34) | 0.85 |
| Miscarriage (<28 weeks gestation) | 23 / 1110 (2.1%) | 18 / 1107 (1.6%) | 1.27 (0.69, 2.35) | 0.43 |
| Stillbirth (≥28 weeks gestation) | 43 / 1087 (4.0%) | 50 / 1089 (4.6%) | 0.86 (0.58, 1.28) | 0.47 |
|  |  |  |  |  |
| *Among Livebirths* |  |  |  |  |
| Mean birthweight (g) | 3112 (550) | 3123 (488) | -10 (-55, 34) | 0.66 |
| Low birthweight (<2500g) | 91 / 1042 (8.7%) | 73 / 1037 (7.0%) | 1.24 (0.92, 1.66) | 0.16 |
| Mean duration gestation (weeks) | 38.5 (3.4) | 38.9 (3.0) | -0.36 (-0.64, 0.09) | 0.009 |
| Preterm birth (<37 completed weeks gestation) | 260 / 1044 (24.9%) | 226 / 1039 (21.8%) | 1.15 (0.98, 1.34) | 0.08 |
| Small-for-gestational age - Oken standard (<10^th^ percentile) [Primary outcome] | 213 / 1042 (20.4%) | 195 / 1037 (18.8%) | 1.09 (0.91, 1.29) | 0.36 |
| Small-for-gestational age - INTERGROWTH-21^st^ standard (<10^th^ percentile) | 183 / 1042 (17.6%) | 186 / 1037 (17.9%) | 0.98 (0.81, 1.18) | 0.82 |
| Infant HIV infection | 22 / 1044 (2.1%) | 22 / 1039 (2.1%) | 0.99 (0.56, 1.77) | 0.98 |
| Neonatal mortality (≤ 28 days) | 30 / 1044 (2.9%) | 21 / 1039 (2.0%) | 1.42 (0.82, 2.46) | 0.21 |
| Infant mortality (≤ 365 days) | 57 / 1044 (5.5%) | 49 / 1039 (4.7%) | 1.15 (0.79, 1.67) | 0.46 |

CI: Confidence interval, HIV: Human immunodeficiency virus, SD: Standard deviation

**Table D.** Exploratory analysis of potential modifiers of the effect of randomized regimen on infant small-for-gestational age birth by Oken standard

|  | Vitamin D  n / N (%) | Placebo  n / N (%) | Relative Risk  (95% CI) | P-value for effect modification |
| --- | --- | --- | --- | --- |
| Overall – all livebirths | 229 / 1070 (21.4%) | 236 / 1091 (21.6%) | 1.09 (0.91-1.29) |  |
| *Subgroups* |  |  |  |  |
| Child sex |  |  |  |  |
| Female | 119 / 534 (22.3) | 108 / 513 (21.1) | 1.07 (0.85, 1.36) | 0.48 |
| Male | 110 / 536 (20.5) | 128 / 578 (22.2) | 0.98 (0.78, 1.23) |  |
| Maternal age |  |  |  |  |
| 18 – 24 years | 27 / 162 (16.7) | 45 / 181 (24.9) | 0.69 (0.45, 1.05) | 0.09 |
| 25 – 34 years | 131 / 637 (20.6) | 127 / 625 (20.3) | 1.05 (0.84, 1.32) |  |
| 35+ years | 71 / 271 (26.2) | 64 / 285 (22.5) | 1.24 (0.91, 1.68) |  |
| Weeks gestation at randomization |  |  |  |  |
| 12-19.9 weeks | 98 / 469 (20.9) | 95 / 456 (20.8) | 1.00 (0.78, 1.29) | 0.74 |
| 20-27 weeks | 131 / 601 (21.8) | 141 / 635 (22.2) | 1.06 (0.85, 1.31) |  |
| Body mass index at randomization |  |  |  |  |
| <25.0 kg/m^2^ | 117 / 502 (23.3) | 114 / 536 (21.3) | 1.10 (0.87, 1.39) | 0.43 |
| ≥ 25.0 kg/m^2^ | 109 / 559 (19.5) | 121 / 547 (22.1) | 0.96 (0.76, 1.22) |  |
| Missing* | 3 / 9 (33.3) | 1 / 8 (12.5) | Not estimable |  |
| Household wealth |  |  |  |  |
| < Median | 113 / 552 (20.5) | 121 / 540 (22.4) | 0.97 (0.77, 1.22) | 0.39 |
| ≥ Median | 116/ 517 (22.4) | 115 / 550 (20.9) | 1.12 (0.88, 1.42) |  |
| Missing | 0 / 1 (0.0) | 0 / 1 (0.0) | Not estimable |  |
| WHO HIV disease stage at randomization |  |  |  |  |
| I | 196 / 935 (21.0) | 194 / 914 (21.2) | 1.04 (0.86, 1.24) | 0.88 |
| II, III, IV | 33 / 135 (24.4) | 42 / 177 (23.7) | 1.07 (0.72, 1.59) |  |
| CD4 T-cell count at randomization |  |  |  |  |
| <350 cells per μL | 39 / 221 (17.7) | 60 / 222 (27.0) | 0.71 (0.50, 1.02) | 0.06 |
| ≥350 cells per μL | 52 / 285 (18.3) | 54 / 287 (18.8) | 1.01 (0.71, 1.45) |  |
| Missing | 138 / 564 (24.5) | 122 / 582 (21.0) | 1.19 (0.95, 1.48) |  |
| Timing of ART initiation |  |  |  |  |
| Before this pregnancy | 104 / 430 (24.2) | 94 / 459 (20.5) | 1.25 (0.97, 1.60) | 0.06 |
| During this pregnancy | 125 / 640 (19.5) | 142 / 632 (22.5) | 0.90 (0.72, 1.13) |  |
| Regimen adherence |  |  |  |  |
| <90% adherence | 124 / 558 (22.2) | 149 / 634 (23.5) | 0.99 (0.79, 1.22) | 0.42 |
| ≥90% adherence | 105 / 512 (20.5) | 87 / 457 (19.0) | 1.12 (0.87, 1.46) |  |

ART: Antiretroviral therapy, CI: confidence interval, HIV: Human immunodeficiency virus, WHO: World Health Organization

**Table E.** Cause of death for infants stratified by treatment group

|  | Vitamin D_3_  N=62 deaths | Placebo  N= 52 deaths |
| --- | --- | --- |
| Pneumonia | 11 | 15 |
| Asphyxia | 12 | 8 |
| Sepsis | 8 | 5 |
| Complications of low birthweight / prematurity | 7 | 2 |
| Sudden infant death | 5 | 3 |
| Meningitis | 3 | 5 |
| Congenital anomaly | 2 | 2 |
| Diarrhea | 1 | 3 |
| Respiratory distress syndrome | 2 | 1 |
| Hemorrhagic disease of the newborn | 1 | 1 |
| Hypoglycemia | 1 | 1 |
| Severe dehydration | 1 | 0 |
| Motor vehicle accident | 1 | 0 |
| Unknown | 7 | 6 |

**Table F.** Effect of maternal vitamin D_3_ supplementation on infant stunting at 1-year of age and secondary infant growth outcomes restricted to singletons

|  | Vitamin D_3_  n / N (%)  No. events / No. at risk (%)  or Mean (SD) | Placebo  No. events / No. at risk^#^ (%)  or Mean (SD) | Relative risk  (95% CI)  or Mean difference* | p-value |
| --- | --- | --- | --- | --- |
| *Infant growth outcomes at 12-months of age* |  |  |  |  |
| Length-for-age z-score (LAZ) | -1.84 ± 1.44 | -1.79 ± 1.45 | -0.06 (-0.19, 0.07) | 0.40 |
| Stunting (LAZ < -2) [Primary outcome] | 391 / 845 (46.3%) | 385 / 833 (46.2%) | 1.01 (0.92, 1.11) | 0.85 |
| Weight-for length z-score (WLZ) | 0.68 ± 1.39 | 0.80 ± 1.38 | 0.11 (-0.03, 0.25) | 0.12 |
| Wasting (WLZ < -2) | 18 / 737 (2.4%) | 13 / 715 (1.8%) | 1.33 (0.66-2.70) | 0.43 |
| Weight-for-age z-score (WAZ) | -0.42 ± 1.16 | -0.30 ± 1.19 | 0.12 (0.00, 0.24) | 0.05 |
| Underweight (WAZ <-2) | 65 / 747 (8.7%) | 50 / 722 (6.9%) | 1.25 (0.88-1.79) | 0.21 |

CI: Confidence interval, LAZ: Length-for-age z-score, SD: Standard deviation, WAZ: Weight-for-age z-score, WLZ: Weight-for length z-score

**Table G.** Exploratory analysis of potential modifiers of the effect of randomized regimen on infant stunting (length-for-age z-score < -2)

|  | Vitamin D  n / N (%) | Placebo  n / N (%) | Relative Risk  (95% CI) | p-value for effect modification |
| --- | --- | --- | --- | --- |
| Overall – all livebirths | 391 / 845 (46.3%) | 385 / 833 (46.2%) | 1.01 (0.92, 1.11) | - |
| *Subgroups* |  |  |  |  |
| Child sex |  |  |  |  |
| Female | 178 / 442 (40.3) | 171 / 408 (41.9) | 0.98 (0.85, 1.14) | 0.45 |
| Male | 229 / 425 (53.9) | 242 / 464 (52.2) | 1.04 (0.93, 1.16) |  |
| Maternal age |  |  |  |  |
| 18 – 24 years | 56 / 127 (44.1) | 59 / 131 (45.0) | 1.04 (0.80, 1.35) | 0.86 |
| 25 – 34 years | 238 / 509 (46.8) | 242 / 505 (47.9) | 0.98 (0.87, 1.11) |  |
| 35+ years | 113 / 231 (48.9) | 112 / 236 (47.5) | 1.02 (0.86, 1.20) |  |
| Weeks gestation at randomization |  |  |  |  |
| 12-19.9 weeks | 177 / 392 (45.2) | 171 / 370 (46.2) | 1.06 (0.92, 1.21) | 0.60 |
| 20-27 weeks | 230 / 475 (48.4) | 242 / 502 (48.2) | 0.99 (0.88, 1.12) |  |
| Maternal body mass index (BMI) at randomization |  |  |  |  |
| <25.0 kg/m^2^ | 184 / 397 (46.4) | 195 / 416 (46.9) | 1.00 (0.87, 1.14) | 0.87 |
| ≥ 25.0 kg/m^2^ | 219 / 461 (47.5) | 214 / 449 (47.7) | 1.01 (0.89, 1.15) |  |
| Missing* | 4 / 9 (44.4) | 4 / 7 (57.1) | Not estimable |  |
| Household wealth |  |  |  |  |
| < Median | 200 / 454 (44.1) | 176 / 434 (40.6) | 1.08 (0.93, 1.25) | 0.29 |
| ≥ Median | 206 / 412 (50.0) | 236 / 437 (54.0) | 0.97 (0.87, 1.09) |  |
| Missing | 1 / 1 (100.0) | 1 / 1 (100.0) | Not estimable |  |
| WHO HIV disease stage at randomization |  |  |  |  |
| I | 353 / 751 (47.0) | 337 / 726 (46.4) | 1.02 (0.92, 1.12) | 0.56 |
| II, III, IV | 54 / 116 (46.6) | 76 / 146 (52.1) | 0.91 (0.73, 1.13) |  |
| CD4 T-cell count at randomization |  |  |  |  |
| <350 cells per μL | 74 / 185 (40.5) | 68 / 172 (39.5) | 1.04 (0.83, 1.32) | 0.79 |
| ≥350 cells per μL | 106 / 236 (44.9) | 101 / 229 (44.1) | 1.05 (0.88, 1.26) |  |
| Missing | 226 / 446 (50.7) | 244 / 471 (51.8) | 0.98 (0.87, 1.11) |  |
| Timing of ART initiation |  |  |  |  |
| Before this pregnancy | 192 / 361 (53.2) | 201 / 392 (51.3) | 1.05 (0.93, 1.19) | 0.61 |
| During this pregnancy | 215 / 506 (42.5) | 212 / 480 (44.2) | 0.99 (0.87, 1.13) |  |
| Regimen adherence |  |  |  |  |
| <90% adherence | 190 / 381 (49.9) | 220 / 437 (50.3) | 0.98 (0.87, 1.12) | 0.58 |
| ≥90% adherence | 217 / 486 (44.7) | 193 / 435 (44.4) | 1.03 (0.91, 1.18) |  |

ART: Antiretroviral therapy, CI: confidence interval, HIV: Human immunodeficiency virus, WHO: World Health Organization

**Table H.** Effect of vitamin D on maternal 25(OH)D concentrations (p-value for difference trajectory between treatment groups: <0.001)

|  | Baseline Mean ± SD | 32 weeks gestation Mean ± SD 25(OH)D ng/mL | 6 weeks postpartum Mean ± SD 25(OH)D ng/mL | 6 months postpartum Mean ± SD 25(OH)D ng/mL | 12 months postpartum Mean ± SD 25(OH)D ng/mL |
| --- | --- | --- | --- | --- | --- |
| Vitamin D_3_ (n=157 participants) | 32.1 ± 9.4 | 49.5 ± 13.7 | 42.0 ± 11.1 | 40.0 ± 10.6 | 37.6 ± 11.0 |
| Placebo (n=160 participants) | 32.6 ± 8.7 | 36.8 ± 10.6 | 29.4 ± 8.6 | 27.1 ± 7.2 | 28.1 ± 6.4 |
| Mean difference in 25(OH)D concentration in ng/mL for vitamin D compared to placebo (95% CI) | -0.5 (-2.5, 1.5) | 12.8 (9.4, 16.1) | 12.6 (10.0, 15.2) | 12.9 (10.3, 15.5) | 9.5 (7.0, 12.0) |
| p-value for mean difference | 0.65 | <0.001 | <0.001 | <0.001 | <0.001 |
|  | | | | | |
|  | Baseline % <30 ng / mL | 32 weeks gestation % <30 ng / mL | 6 weeks postpartum % <30 ng / mL | 6 months postpartum % <30 ng / mL | 12 months postpartum % <30 ng / mL |
| Vitamin D_3_ (n=157 participants) | 40.8% | 7.6% | 12.7% | 20.6% | 30.4% |
| Placebo (n=160 participants) | 39.4% | 23.8% | 57.9% | 64.5% | 64.4% |
|  | | | | | |
|  | Baseline % <20 ng / mL | 32 weeks gestation % <20 ng / mL | 6 weeks postpartum % <20 ng / mL | 6 months postpartum % <20 ng / mL | 12 months postpartum % <20 ng / mL |
| Vitamin D_3_ (n=157 participants) | 10.8% | 2.8% | 4.6% | 2.1% | 2.9% |
| Placebo (n=160 participants) | 4.4% | 5.7% | 12.3% | 17.2% | 7.9% |

25(OH)D: 25-hydroxyvitamin D, CI: Confidence interval, SD: Standard deviation

**Table I.** Effect of vitamin D on infant 25(OH)D concentrations at 6 weeks, 6 months and 12 months of age (p-value for difference in 25(OH)D trajectory between treatment groups: <0.001)

|  | Infant 6 weeks  Mean ± SD 25(OH)D ng/mL | Infant 6 months  Mean ± SD 25(OH)D ng/mL | Infant 12 months Mean ± SD 25(OH)D ng/mL |
| --- | --- | --- | --- |
| Vitamin D_3_ (n=151 infants) | 29.8 ± 10.1 | 34.6 ± 9.3 | 31.6 ± 8.8 |
| Placebo (n=156 infants) | 16.6 ± 9.4 | 30.4 ± 8.9 | 29.6 ± 7.3 |
| Mean difference in 25(OH)D concentration in ng/mL for vitamin D compared to placebo (95% CI) | 13.2 (10.6, 15.9) | 4.2 (1.6, 6.8) | 2.0 (-0.4, 4.4) |
| p-value for mean difference | <0.001 | 0.002 | 0.10 |
|  | | | |
|  | Infant 6 weeks postpartum % <30 ng / mL | Infant 6 months postpartum % <30 ng / mL | Infant 12 months postpartum % <30 ng / mL |
| Vitamin D_3_ (n=151 infants) | 49.5% | 25.6% | 48.9% |
| Placebo (n=156 infants) | 92.9% | 52.5% | 42.5% |
|  | | | |
|  | Infant 6 weeks postpartum % <20 ng / mL | Infant 6 months postpartum % <20 ng / mL | Infant 12 months postpartum % <20 ng / mL |
| Vitamin D_3_ (n=151 infants) | 15.0% | 4.4% | 5.8% |
| Placebo (n=156 infants) | 61.6% | 10.1% | 11.1% |

25(OH)D: 25-hydroxyvitamin D, CI: Confidence interval, SD: Standard deviation

**Figure A**. Mean and standard deviation of length-for-age z-score (LAZ) from 6 weeks to 52 weeks of child age for vitamin D_3_ (red line) and placebo groups (blue line) (p-value for difference in LAZ trajectory between randomized groups: 0.19).


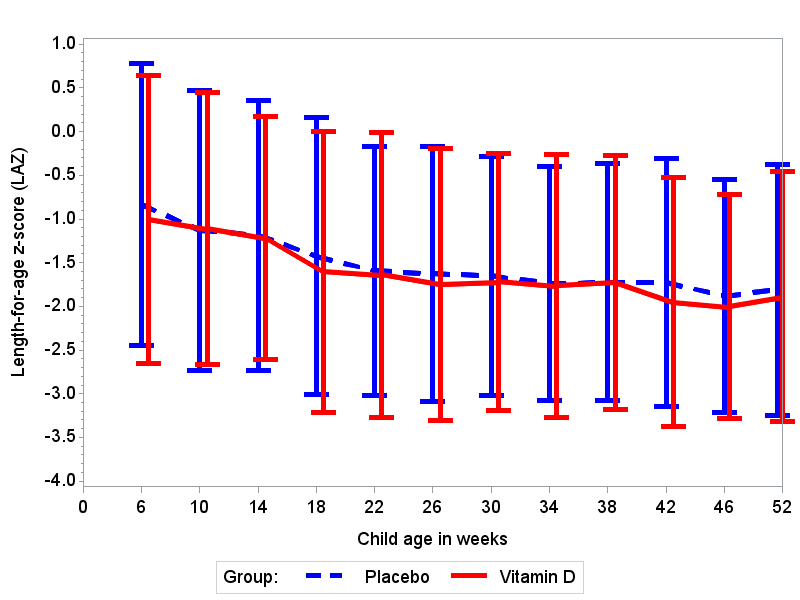


LAZ: Length-for-age z-score

**Figure B**. Mean and standard deviation child weight-for-length z-score (WLZ) for vitamin D_3_ (red line) and placebo groups (blue line) from 6 weeks to 52 weeks of child age (p-value for difference in WLZ trajectory between randomized groups: 0.67).


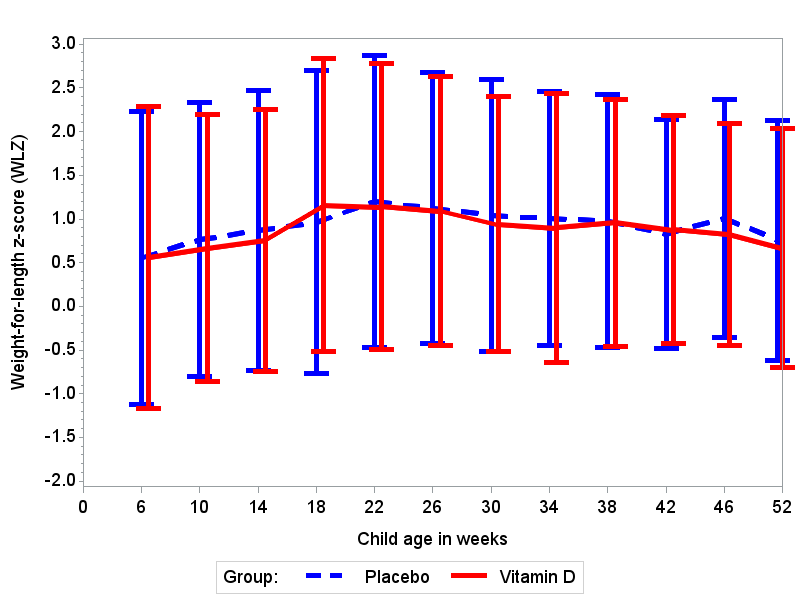


WLZ: Weight-for-length z-score

**Figure C**. Mean and standard deviation child weight-for-age z-score (WAZ) for vitamin D_3_ (red line) and placebo groups (blue line) from 6 weeks to 52 weeks of child age (p-value for difference in WAZ trajectory between randomized groups: 0.60).


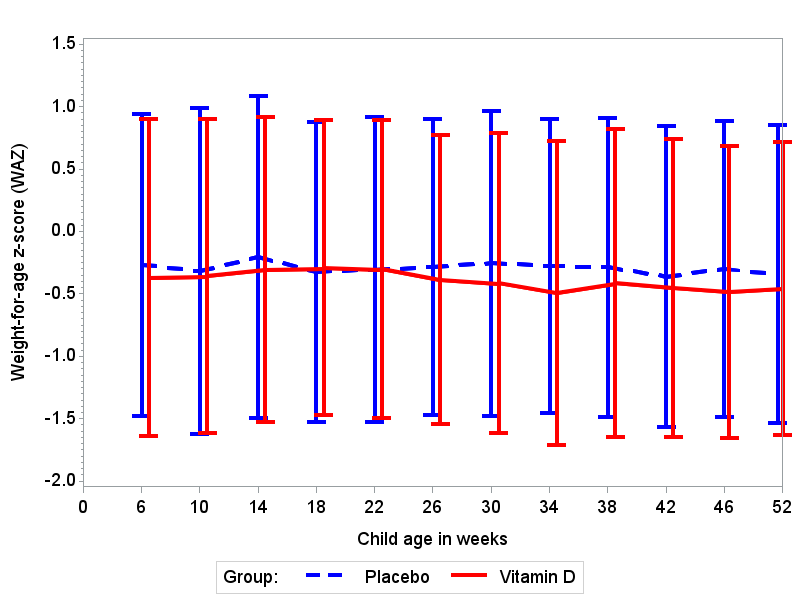


WAZ: Weight-for-age z-score
